# Involvement of Histone Acetylation of Sox17 and Foxa2 Promoters during Mouse Definitive Endoderm Differentiation Revealed by MicroRNA Profiling

**DOI:** 10.1371/journal.pone.0027965

**Published:** 2011-11-23

**Authors:** Shijun Fu, Qi Fei, Hua Jiang, Shannon Chuai, Song Shi, Wen Xiong, Lei Jiang, Chris Lu, Peter Atadja, En Li, Jianyong Shou

**Affiliations:** China Novartis Institutes for BioMedical Research, Shanghai, China; Baylor College of Medicine, United States of America

## Abstract

Generation of hepatocyte from embryonic stem cells (ESCs) holds great promise for hepatocyte replacement therapy to treat liver diseases. Achieving high efficiency of directed differentiation of ESCs to hepatocyte is of critical importance. Previously, Wnt3a has been reported to promote Activin A-induced human definitive endoderm (DE) differentiation, the early stage of hepatocyte differentiation. However, the underlying molecular mechanisms are not clear. Growing evidence demonstrated that microRNAs (miRNAs) are key regulators involved in various important biological processes including the regulation of stem cell differentiation. In the present study, we profiled genome wide miRNA expression during Wnt3a and Activin A induced mouse DE differentiation. We uncovered distinct miRNA expression patterns during DE differentiation with the identification of a subset of miRNAs whose expression is synergistically regulated by Wnt3a/Activin A treatment at different stages of DE differentiation. Forced expression of a pool of such synergistically regulated miRNAs alone could partially promote DE differentiation, indicating a regulatory role of them. Using TargetScan and GeneGO pathway analyses, the synergistically regulated miRNAs are predicted to regulate key pathways involved in DE differentiation; among them includes the regulation of histone acetylation. Consistently, Wnt3a and Activin A treatment increased global histone acetylation which can be partially mimicked by over expression of the pooled miRNAs. Chromatin IP (ChIP) experiments demonstrated that the promoter regions of Sox17 and Foxa2 are subjected to histone acetylation regulation. Administration of Hdac inhibitors greatly augmented DE differentiation. Our data uncovered a novel epigenetic mechanism of Wnt3a and Activin A induced DE differentiation, whereby the treatment of growth factors induced histone acetylation at least in part by the regulation of miRNA expression.

## Introduction

Diseases like cirrhosis and cancer caused liver injuries usually lead to liver dysfunction and/or organ failure. Liver transplantation is so far the only effective treatment for liver cancer/failure or other end stage liver diseases [1]. Shortage of donor organs has limited its clinical applications. Treating liver cancer and other liver diseases remains a major unmet medical need. Hepatocyte transplantation has emerged as a promising alternative to liver transplantation for treating liver diseases. However, such cell replacement therapy is greatly hindered by the limited availability of functional human hepatocyte suitable for transplantation. Hepatocytes are also widely used in drug toxicity screen. Therefore, establishing and understanding the mechanism of hepatogenic differentiation paradigm is not only scientifically interesting, but also clinically important.

The potential of ESCs to differentiate into cell types of a variety of organs has generated much excitement for regenerative medicine. Of great interests are DE derived organs, such as liver and pancreas. Although several hepatocyte differentiation platforms have been reported [2]–[5], the underlying mechanisms are not fully understood. Activin A, the member of transforming growth factor (TGF)-beta super-family, is known to play key roles in directing ESC differentiation into DE, the early stage of hepatocyte differentiation [6]. In addition, other factors such as members of Wnt signaling pathway also play important roles in DE formation; disruption of the Wnt signaling pathways prevents endoderm formation during mouse embryo development [7], [8]. *In vitro,* Wnt3a signaling is also reported to be required for efficient differentiation of human ESCs to functional hepatic endoderm induced by Activin A, suggesting Wnt3a can improve the efficiency of Activin A-induced DE differentiation [9]. However, the detailed mechanisms for the synergistic effect are not understood.

microRNAs (miRNAs) are endogenous, short (about 22-nucleotide) noncoding RNAs, known to regulate the expression of target genes by degrading the target mRNA transcripts and/or by inhibition of mRNA translation [10]. Up to 30% of human genes are predicted to be regulated by miRNAs [11], suggesting a central role for miRNAs in the regulation of gene expression at post-transcriptional level. *In vivo*, recent reports revealed that embryonic development was accompanied by dynamic expression of miRNAs. miRNAs such as lin-4 and let-7 are critical for temporal control of larval development in c.elegans [12], [13]. In mouse preimplantation development, a set of miRNAs are tightly regulated during trophectoderm specification [14]. *In vitro*, characterizations of miRNAs expression in mouse and in human ESCs and ESCs-derived embryoid bodies have been recently published and revealed two highly-expressed clusters [15]–[17]. Furthermore, DE specific miRNAs have been identified in human ESC-derived endoderm cells [18], [19]; suggesting that miRNAs play important roles in DE differentiation.

In this report, we performed a genome-wide analysis of miRNA expression in Activin A and Wnt3a-treated mouse ESCs during the different stages of DE differentiation to identify candidate miRNAs likely to be involved in Wnt3a and Activin A induced DE formation. Our analysis exhibited a distinct miRNA expression fingerprint. Furthermore, we found that forced expression of a subset of synergistically regulated miRNAs could partially mimic the roles of Wnt3a and Activin A. Pathway analyses also revealed the involvement of histone acetylation in Activin A/Wnt3a-driven DE differentiation, which is further confirmed by treating the cells with small molecular weight Hdac inhibitors as well as ChIP experiments. Our study established a regulatory cascade from extracellular cytokine treatment to miRNA expression to histone modification in cell nuclei during DE differentiation.

## Materials and Methods

### Cell culture

The mouse ES TCL-1 cells were derived from 129/SvJae mice using the same protocol as previously described [20], which is according to national and international guidelines. This procedure was approved by the Animal Ethics Committee at Novartis Institutes for Biomedical Research in Cambridge (approved animal protocol 09 DMP 072). The passage number used here ranged from 10 to 20. mESCs were maintained on mitomycin C-treated MEF feeder layers in DMEM medium supplemented with 15% FBS, 1 mM L-glutamine, 1 mM nonessential amino acids, 0.1 mM beta-mercaptoethanol, 1% Penicillin/streptomycin (all from Invitrogen, Carlsbad, USA), and 1000 unit/ml LIF (Millipore, Billerica, USA) under a standard gas atmosphere of humidified air/5% CO2.

### Definitive endoderm and hepatocyte differentiation

Mouse ESCs were maintained on MEF feeder cells until they reached about 80% confluence. Prior to differentiation, mouse ESCs were passaged onto gelatin-coated plates for 1 hour to remove feeders, and afterwards, ESCs were seeded at 80000 cells per well on collagen-coated 6 well plate. After overnight culture, cells were treated with 100 ng/ml Activin A (R&D system, Minneapolis, USA), or 50 ng/ml Wnt3a (R&D system, Minneapolis, USA), or 100 ng/ml Activin A plus 50 ng/ml Wnt3a (R&D system, Minneapolis, USA) in RPMI 1640 (Invitrogen, Carlsbad, USA) supplemented with 1XB27 and 1X sodium butyrate (Invitrogen, Carlsbad, USA) for 3 to 5 days for endoderm differentiation. For hepatic induction, after 5 days of Activin A plus Wnt3a treatment, the differentiated cells were cultured in hepatocyte culture medium (HCM) (Lonza, Basel, Switzerland) containing 30 ng/ml FGF4 (R&D system, Minneapolis, USA) and 20 ng/ml BMP2 (R&D system, Minneapolis, USA) for 5 days, then the differentiated cells were further treated with 20 ng/ml HGF (R&D system, Minneapolis, USA) in HCM for maturation. Cells cultured in the same medium without growth factors were used as the negative control. Through the entire culture period, medium was replenished every day.

To assess the effects of Hdac inhibition on DE differentiation, the mouse ESCs were treat the same as above, except TSA (Sigma, St. Louis, USA) was added at the final concentration of 1–10 nM to the differentiation medium. DMSO alone added samples were used as the negative control. Cells were harvested for RNA isolation and IF analyses at different stages of differentiation.

### RNA isolation and miRNAs profiling

ESCs were treated with 100 ng/ml Activin A, or 50 ng/ml Wnt3a, or 100 ng/ml Activin A plus 50 ng/ml Wnt3a, and the samples were collected at 1 day, 3 days, and 5 days after treatment, respectively. Cells treated with the same medium without growth factors were used as the negative controls for each time point. All samples were lysed in Trizol and RNA was extracted using the procedure recommended by the manufacture (Invitrogen, Carlsbad, USA) and DNA containment was removed using DNase digestion. RNA quality was confirmed by Agilent bioanalyzer 2100.

Agilent miRNA arrays were performed in one color according to the manufacturer's instructions at Shanghai Biochip Corporation, China (Agilent Technologies, Santa Clara, USA). Data were acquired using Agilent Feature Extraction software version 9.5.3.1. To normalize the data across different arrays, quantile normalization method was applied and all further analyses were carried out using the normalized and background subtracted intensity values [21], [22]. Further data analyses were performed by using GeneSpring GX 10.0, cluster 3.0, treeview and R. In brief, the qualities of raw data were examined by hierarchal clustering and principal component analysis (PCA) (Figure S1). Differentially expressed miRNAs were identified by one-way ANOVA test under following criteria (FDR<0.05, and Log2 difference>1or <−1, respectively). The synergistically regulated miRNAs were identified using the following criteria: the miRNAs were up- or down- regulated at least 1.5 fold in Activin A plus Wnt3a treated samples compared to Activin A treated samples, while, the miRNAs were not significantly regulated in Wnt3a alone treated samples. The differentially expressed miRNAs were clustered using cluster 3.0 and displayed using Treeview. For miRNAs expression finger print analysis, the differentially expressed miRNAs were re-normalized by using Z-score normalization in spotfire, and then further clustered by 9×9 SOM in Genespring. After that, for each sample, these miRNAs were averaged in each cluster and presented by heatmap which respond to each cluster in SOM. For pathway analysis, first, we predicted target genes of synergistically regulated miRNAs using TargetScan version 5.1 and Miranda, and further used these genes as an input for pathway analysis using GeneGO software [23]. All data in our study is MIAME compliant and that the raw data has been deposited in the National Center for Biotechnology Information Gene Expression Omnibus repository (GEO accession no. GSE29093).

### Immunofluorescence (IF)

Cells were fixed in 4% paraformaldehyde in phosphate-buffered saline (PBS) at room temperature for 20 minutes and treated with 0.5% Triton X-100 for 30 min, then washed with PBS for three times, The fixed cells were blocked with PBS containing 3% bovine serum albumin at room temperature for 1 hour. Cells were incubated with primary antibodies at 4°C overnight. Normal rabbit serum was used as a negative control, and no fluorescence was observed in the negative controls. The primary antibodies against Sox17 (R&D system, Minneapolis, USA) and Foxa2 (Millipore, Billerica, USA) were diluted at 1∶300, antibodies against Albumin (ABcam, Cambridge, England) and AFP (R&D system, Minneapolis, USA) was diluted at 1∶200, respectively. After washes with PBS three times, FITC-conjugated or TRITC-conjugated secondary antibody (Invitrogen, Carlsbad, USA) diluted at 1∶1000 was added and incubated at 37°C for 1 hour. Then 1 ug/ml DAPI was used to stain the cell nuclei.

### Real-time RT-PCR (RT-qPCR)

Real-time RT-PCR analysis was performed on an ABI Prism 7900 Sequence Detection System using the SYBR Green PCR Master Mix and miRNA Taqman Master Mix (Applied Biosystems, Foster City, USA), respectively. The PCR reaction was performed as described [24]. The relative expression of each gene was normalized against Gapdh, and the relative expression of each miRNAs was normalized against U6 snRNA, respectively. The primers and miRNAs probe number used for the quantitative RT-PCR are shown in Table S1.

### miRNAs over-expression

In order to over-express miRNAs in differentiating ESCs, six synthetic mature miRNAs (Invitrogen), mir-181c/338-5p/222/196a/196b/let-7e, were pooled together equivalently. Cel-miR-67, which has been confirmed to have minimal sequence identity with miRNAs in human, mouse and rat, was used as a negative control. The final concentration of the pooled miRNAs and the control miRNA (Cel-67) is 20 uM. Mono-layer ESCs were treated with 100 ng/ml Activin A for three days in collagen coated 6-well plate, then transfect with 10 ul pooled miRNAs by using 5 ul RNAMAXi reagent (Invitrogen, Carlsbad, USA). The transfected cells were allowed for further differentiation for 2 more days. At day 5, the differentiating cells were transfected one more time with 10 ul of pooled miRNAs the same as above. 5 days after the initial transfection, all the samples were collected and analyzed for DE marker expression. The detailed protocol was described in Methods S1.

### Inhibition of miRNA expression by synthetic miRNA inhibitors

In order to confirm the roles of the regulatory miRNAs in Activin A and Wnt3a induced DE cell differentiation and histone acetylation level, we over-expressed pooled miRNA inhibitors in differentiating ESCs. The synthetic miRNA inhibitor experiment was carried out essentially the same as that of the pooled miRNA over-expression experiment. Six synthetic mature miRNA inhibitors including mir-181c/338-5p/222/196a/196b/let-7e were pooled together equivalently. Cel-miR-67 inhibitor was used as a negative control. All miRNAs inhibitors were synthesized by Invitrogen (Carlsbad, USA). At the 5th day of differentiation, the samples were collected and analyzed on DE markers and histone acetylation levels.

### Western blot analysis

Cells were lysed in 1XRIPA lysis buffer in the presence of protease inhibitor mixture (Roche, Basel, Switzerland). To determine protein concentration, the lysates were measured using a Bio-Rad BCA protein assay (Bio-Rad, Richmond, USA), and resolved on a 10% SDS-PAGE gel, then transferred to a nitrocellulose membrane (Invitrogen, Carlsbad, USA). After blocking with Superblock T20 Blocking buffer (Thermo scientific, Waltham, USA), the membrane was incubated with a primary antibody overnight at 4°C and then with a secondary antibody conjugated with alkaline phosphatase (1 h at room temperature), the signal was detected by using a chemiluminescence method. The following polyclonal primary antibodies were used: anti-human Sox17 (1∶1000, R&D system, Minneapolis,USA), anti-H3K9Ac (1∶1500, Cell Signaling, Danvers, USA), anti-H3K27Ac (1∶1500, Cell Signaling, Danvers, USA), anti-Tubulin (1∶10000, Santa Cruz Biotechnology,California, USA).

### Chromatin Immunoprecipitation PCR (ChIP-PCR)

Mouse ESCs were cultured in differentiation medium for 2 days as mentioned above. The cells were then treated with fresh culture medium containing 1% formaldehyde for 10 minutes, and washed twice with ice cold PBS containing protease inhibitors. The cell pellets were then re-suspended in SDS lysis buffer also containing protease inhibitors (200 µl lysis buffer for every 1*10^6^ cells) and incubated on ice for 10 minutes. Cell lysate was sonicated (15w for 10 seconds for three times) to shear DNA to lengths between 200 and 1000 base pairs. The next ChIP experimental procedures were performed following the protocol of chromatin Immunoprecipitation (ChIP) Assay Kit (Millipore17–295) using the antibody against H3K9Ac (Cell Signaling, Danvers, USA). The eluted DNA was used to assess the level of acetylated H3K9 recruited to Sox17 and Foxa2 promoters by PCR and qPCR. For each gene, the primers were designed based on the sequence 1 kb to 2 kb upstream of transcript initiation site. The sequences were listed in Table S1.

## Results

### Wnt3a improves the efficiency of Activin A-induced DE formation from mouse ESCs

**Figure 1:**
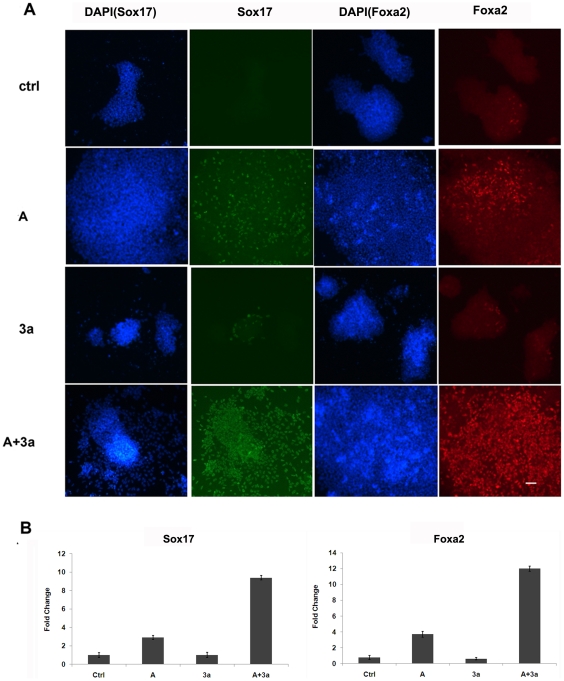
Wnt3a improves the efficiency of Activin A induced DE differentiation from mouse ESCs. The mouse ESC line, TCL-1 cells, were treated with 100 ng/ml Activin A, 50 ng/ml Wnt3a, or 100 ng/ml Activin A plus 50 ng/ml Wnt3a, respectively. The differentiated cell samples were collected 5 days after treatment for DE marker analyses by using IF and RT-qPCR. A. IF staining of Sox17 and Foxa2 expression in differentiated mouse ESCs treated with different growth factors. Scale bar: 50 uM. B. RT-qPCR examination of the expression of DE markers, Sox17 and Foxa2. Data are expressed as mean fold change ± SD. A: Activin A; 3a:Wnt3a.

DE has been successfully derived *in vitro* from mouse ESCs in the culture condition of low serum and high concentration of Activin A [25]. It has been shown previously that Wnt3a could improve the efficiency of Activin A-induced DE differentiation from human ESCs [9]. We hypothesized that Wnt3a might also improve the efficiency of Activin A-induced DE formation in mouse ESCs. To test this hypothesis, we treated mouse ESCs with Activin A either in the presence or absence of Wnt3a. After treatment in serum-free culture medium for 5 days, cells were analyzed for their expression of specific DE markers (Sox17 and Foxa2). Immunofluorescence (IF) staining revealed that about 80% of the cells treated with Activin A and Wnt3a expressed Sox17 or Foxa2, while only about 40% of the cells were Sox17 or Foxa2 positive when treated with Activin A alone (Figure 1A). Wnt3a alone did not affect DE differentiation. RT-qPCR further confirmed that the expression of Sox17 and Foxa2 was significantly increased upon co-treatment with Activin A and Wnt3a, while addition of Wnt3a alone did not induce DE formation from mouse ESCs (Figure 1B). Activin A and Wnt3a co-treatment did not significantly induce the expression of Nestin, Cdx2, Bmp4, or mT (data not shown), suggesting that most of the differentiated cells were not mesoderm or ectoderm cells. Together, our data indicated that Wnt3a can augment mouse ESC derived DE formation by acting synergistically with Activin A.

### DE cells induced by Activin A and Wnt3a are competent for hepatocyte differentiation

**Figure 2:**
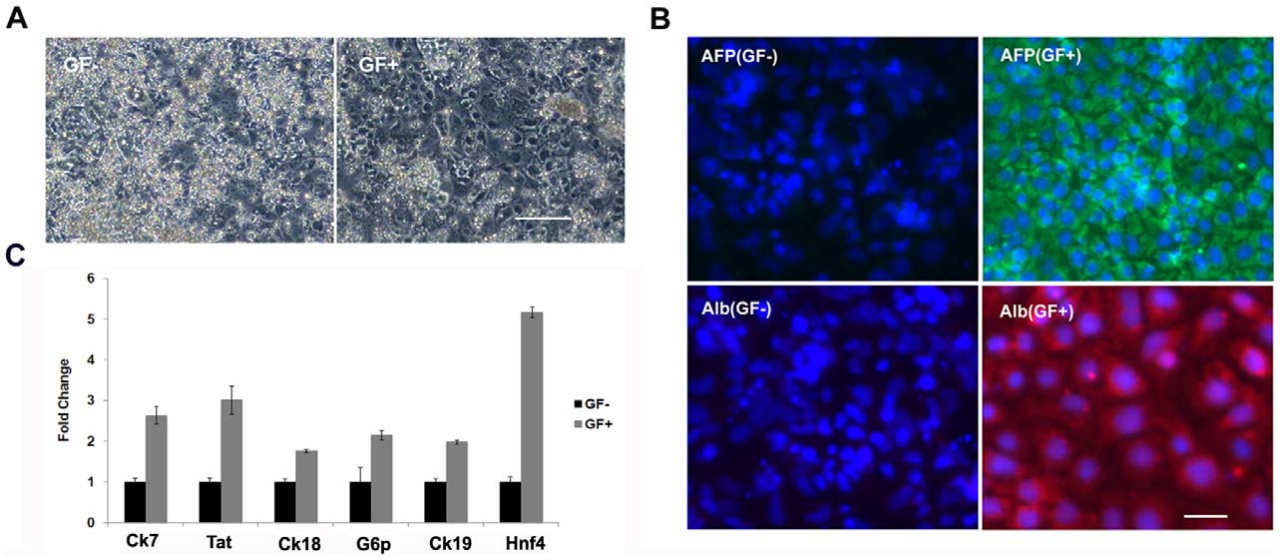
Hepatic differentiation of Activin A and Wnt3a induced mouse DE. Mouse DE cells induced by Activin A and Wnt3a were treated with growth factors (GFs) for further differentiation into hepatocytes for 8 days. A. Morphology of differentiated cells with or without GFs. Please note that the GF treated cells demonstrated typical hepatocyte like morphology. Scale bar: 50 uM. B. IF staining of AFP and Albumin (Alb) in differentiated cells. Scale bar: 20 uM. C. RT-qPCR examination of hepatocyte marker expression in the differentiated cells. Data are expressed as mean fold change ± SD. GF: 30 ng/ml FGF4, 20 ng/ml BMP2, 30 ng/ml HGF.

It has been shown that ESC-derived DE could further differentiate into hepatocytes by treatment with FGFs, BMP and HGF sequentially. We next addressed whether the mouse DE cells induced by Activin A and Wnt3a are competent of hepatocyte differentiation. The DE cells induced by Wnt3a/Activin A were further treated with FGF4 and BMP2 for hepatic initiation, and subsequently with HGF for hepatic maturation. After total 8 days of differentiation, the cells became more flattened morphologically (Figure 2A); most of the differentiated cells expressed AFP and Albumin by IF analysis (Figure 2B), indicating that these cells have differentiated into hepatocyte-like cells. We further confirmed their hepatic cell identity by examining the expression of other known hepatocyte markers using RT-qPCR. We found that, under the current differentiation conditions, the differentiated cells are positive for various hepatocyte markers including Ck7, Ck18, Ck19, Tat, G6p, and Hnf4 (Figure 2C). These results suggested that mouse DE cells induced by Activin A and Wnt3a were capable of differentiating into hepatocytes.

### Characterization of miRNA expression fingerprint during DE differentiation

We have shown that Wnt3a could act synergistically with Activin A to induce mouse DE formation as they did to human ESCs [18], [19]. Nevertheless, the underlying molecular mechanism is unknown. Since it has been well established that miRNAs are involved in various development and differentiation processes, we asked whether the synergy between Wnt3a and Activin A in DE differentiation involves the regulation of miRNAs. Previous studies had uncovered some miRNAs expressed by human ESC-derived DE cells [18], [19]. We examined the expression of two of them, mir-125a-5p and mir-let-7e, during mouse DE differentiation. The result shows that they were also regulated by Activin A and Wnt3a in mouse ESC derived DE differentiation (Figure S2). These two miRNAs do not show significant synergistic expression pattern upon Activin A and Wnt3a treatment.

**Figure 3:**
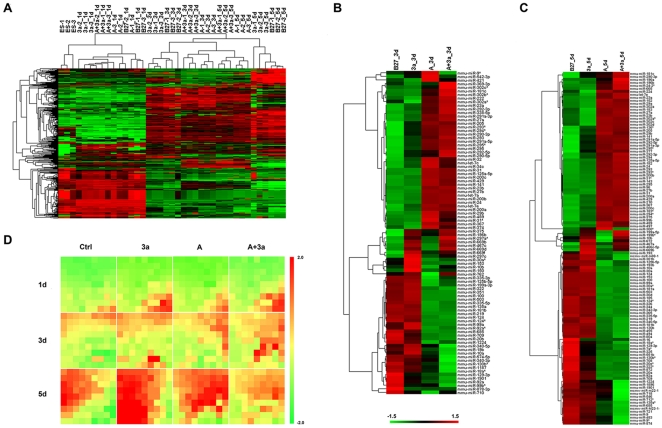
Differentially expressed miRNAs during DE differentiation from mouse ESCs. Mouse ESCs were treated with 100 ng/ml Activin A, 50 ng/ml Wnt3a, or 100 ng/ml plus 50 ng/ml Wnt3a, and the samples were collected and profiled for miRNA expression at 1 day, 3 days, and 5 days after treatment. The differentially expressed miRNAs were identified by using ANOVA test under FDR <0.05. A. 386 differentially expressed miRNAs in all samples were presented using hierarchal clustering analysis. The miRNAs expression values were normalized by Z-score normalization, and the hierarchal clustering was performed in Cluster3.0 using average linkage with Euclidean being used as the distance metrics. B, C. Hierarchal clustering of 90 and 118 differentially expressed miRNAs under the cutoff as FDR <0.05 and fold>2 or <0.5 in 3 day and 5 day samples, respectively. D. 386 differentially expressed miRNAs identified by ANOVA were analyzed in Genespring using 9×9 SOM methods with Euclidean being used as the distance metrics. Heatmap presented average expression pattern of the miRNAs in each cluster from SOM in different treatment groups. The position of each cluster was presented as Cluster (Cij), i means row; j means column. For example, C32 means the cluster at the third row and second column. Please note the distinct expression fingerprint of miRNAs during DE differentiation. The differentially expressed miRNAs for each time point were listed in Table S2, S3, and S4, respectively. A: Activin A; 3a:Wnt3a.

To gain a comprehensive understanding of the effects of Wnt3a and Activin A on miRNAs expression, we performed global miRNA expression profiling using microarray. We treated mouse ESCs with Activin A, Wnt3a or Activin A plus Wnt3a, and collected the RNA samples at 1 day, 3 days and 5 days after treatment, representing different stages of DE differentiation (namely, DE initiation, DE progression, and DE maturation, respectively). We used the Agilent miRNA microarrays for the profiling study. ANOVA test showed that 386 miRNAs were differentially expressed under the cutoff of FDR<0.05 among all the treatment groups during Wnt3a/Activin A-induced DE differentiation. The overall expression pattern of the differentially expressed miRNAs was demonstrated by hierarchal clustering analysis (Figure 3A). We further identified the differentially regulated miRNAs respect to the various stages of differentiation. At DE initiation, Only 4 miRNAs were significantly regulated under the cutoff as FDR<0.05 and fold>2 or <0.5. While differentiation proceeds, increasing number of miRNAs (90 miRNAs and 118 miRNAs for DE progression and maturation, respectively) were differentially expressed (Figure 3B, C). In order to confirm our microarray data, we selected a subset of miRNAs and tested them by using Taqman RT-qPCR analysis using independent RNA samples (Figure S3). The RT-qPCR results were in good agreement with our microarray findings although the fold changes were usually higher from the array data due to the inherent technical difference between the two platforms.

To further explore miRNA expression pattern during DE differentiation, differentially expressed miRNAs were analyzed by using self-organizing map (SOM) and presented using a heatmap visualization (Figure 3D). The results revealed that at DE initiation the expression patterns of miRNAs of different treatment groups were very similar; when differentiation proceeds, we observed distinct miRNA expression patterns. The control and Wnt3a alone treated samples shared a very similar miRNA expression pattern, which was in agreement with that Wnt3a alone had little effect on DE differentiation. However, Activin A and Activin A plus Wnt3a treatment groups induced dramatic miRNA expression changes.

We next examined the molecular functions and the biological relevance of some of the differentially expressed miRNAs within various clusters of the SOM. mir-335 family members mir-335-3p and mir-335-5p, two of the most significantly regulated miRNAs, belong to cluster 32(C32) and cluster 41(C41), suggesting that they may be mainly driven by spontaneous differentiation (Figure 3D). On the other hand, some known miRNAs involved in DE-related tissue development fall into distinct clusters likely driven by Wnt3a/Activin A treatment (Figure 3D). For examples, mir-218 (C46) is dynamically expressed in developing liver and pancreas, which targets Hepatocytes Nuclear Factor-6 (HNF-6/OC-1) and OC-2 to regulate liver and pancreas development [26]. mir-375 (C48) is selectively expressed in pancreas, which targets 3′-phosphoinositide dependent protein kinase-1 and regulates glucose induced biological responses in pancreatic beta-cells [27].

### Identification of miRNAs synergistically regulated by Wnt3a and Activin A

**Figure 4:**
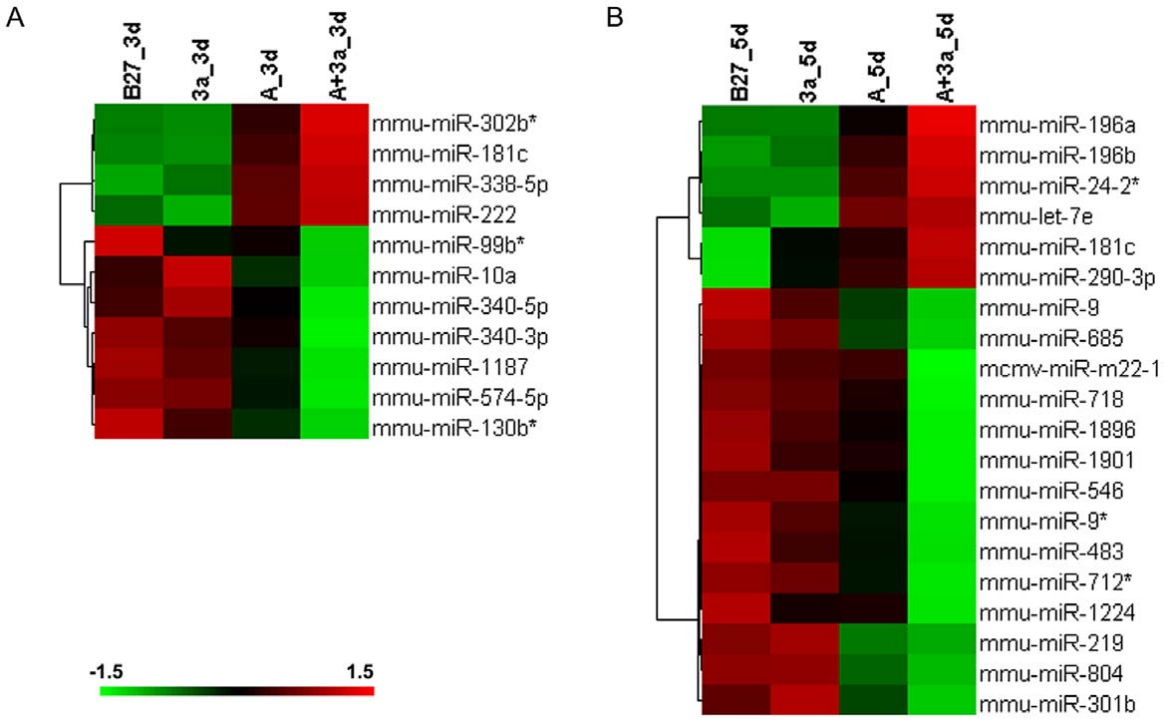
Synergistically regulated miRNAs by Wnt3a and Activin A during mouse DE formation. Synergistically regulated miRNAs were identified using the criteria stated in the results section. The synergistically expressed miRNAs identified at day 3 (A) and day 5 (B) was presented using the heatmap visualization. No miRNA was found to be synergistically regulated at day 1 samples under current cutoffs. A: Activin A; 3a:Wnt3a.

Some of the differentially expressed miRNAs might play regulatory roles in DE differentiation, i.e., they play instructive roles directing DE differentiation rather than being molecular markers resulted from DE differentiation. We hypothesized that the miRNAs showing synergistic pattern upon Wnt3a/Activin A treatment might have a higher probability of being regulatory. We applied the following criteria to select the synergistic miRNAs: the miRNAs were up- or down- regulated at least 1.5 fold in Activin A plus Wnt3a treated samples compared to Activin A treated samples, while, they were not significantly regulated in Wnt3a alone treated samples. Under such criteria, at DE initiation, no miRNAs were synergistically regulated. While, at DE progression 10 miRNAs were synergistically regulated. In addition, mir-10a which was down regulated in Activin A treated samples was further down regulated in Activin A plus Wnt3a treated samples, while it was up-regulated in Wnt3a treated sample. We considered it as one synergistically regulated miRNA as well. Figure 4A displays the expression pattern of these synergistically regulated miRNAs at DE progression. Among these miRNAs, some of them, such as mir-222, has been reported to regulate endoderm differentiation [18]. In addition, we also identified some miRNAs, which were not previously reported to regulate ESC derived endoderm differentiation, such as mir-338-5p and mir-340-3p. The potential roles of these miRNAs on promoting DE differentiation warrant future investigation. At DE maturation, 20 miRNAs were found to be synergistically regulated (Figure 4B). Very little synergistically regulated miRNAs were overlapped between DE progression and maturation. Strikingly, many of these synergistically regulated miRNAs were also found to be regulated in hESCs derived DE [18] such as mir-196a, mir-196b, and miR-24-2*, suggesting that the roles of miRNAs on DE differentiation might be conserved between human and mouse.

**Figure 5:**
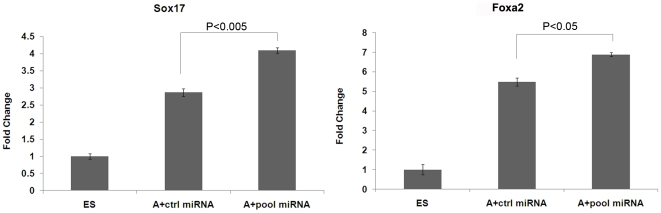
Over-expression of pooled miRNAs partially increases the expression of DE markers. Mouse ESCs were treated with 100 ng/ml Activin A for three days, then transfected with pooled miRNAs to allow further differentiation for 2 more days. Cel-miR-67 was use as a negative control. The expression of two DE markers Sox17 and Foxa2 was analyzed by RT-qPCR. The expression of these two markers was significantly increased after treating with pooled miRNAs as compared to the control cells (*P* = 0.003 for Sox17, and *P* = 0.028 for Foxa2). Data are expressed as mean fold change ± SD. A: Activin A.

MiRNAs have been reported to play roles in promoting and directing cell differentiation. For example, miR-17-5p, miR-20a, miR-93, and miR-106a, were found to be differentially expressed in developing mouse embryos and function to control differentiation of stem cells [28]. In light of this, we decided to test whether forced expression of a subset of miRNAs we identified in the current study could themselves affect the differentiation process. We over-expressed a pool of miRNAs consisting mir-181c/338-5p/222/196a/196b/let-7e at 3 days of differentiated ES cells and then examined the DE markers 2 days later. The over-expressed miRNA level was confirmed by using RT-qPCR (Figure S4). The result revealed that all these miRNAs were significantly over-expressed in differentiated cells. Moreover, the RT-qPCR results indicated that the expression of Sox17 and Foxa2 were significantly increased upon pooled miRNA over-expression (Figure 5). The data suggested that forced expression of these pooled miRNA could facilitate Activin A-induced DE differentiation in a similar way to Wnt3a.

### Pathway analyses of synergistically regulated miRNAs

In order to further explore the functions of the synergistically regulated miRNAs, we predicted the putative target genes for these miRNAs by using TargetScan 5.0 software, and then performed functional enrichment test using GeneGO software. At DE progression, several pathways were significantly enriched (Table 1). Among the enriched pathways, TGF-beta signaling pathway was the most significantly enriched one, consistent with the known roles of SMAD2/3 in regulating endoderm differentiation [29]. In addition, a few other known pathways involved in classical endoderm differentiation such as HGF, IGF were also significantly enriched, suggesting that they may also be subject to miRNA regulation during Wnt3a and Activin A-induced DE differentiation.

**Table 1:** The 10 most significantly enriched pathways at DE progression stage.

Name	pValue	Network objects
Development_TGF-beta receptor signaling	3.77E–12	19/50
Development_Role of HDAC and calcium/calmodulin-dependent kinase (CaMK) in control of skeletal myogenesis	1.86E–11	19/54
Cytoskeleton remodeling_TGF, WNT and cytoskeletal remodeling	2.08E–11	27/111
Cell adhesion_Ephrins signaling	6.09E–09	15/45
Development_Slit-Robo signaling	1.93E–08	12/30
Stem cells_Early embryonal hypaxial myogenesis	3.10E–08	13/37
Development_IGF-1 receptor signaling	4.17E–08	15/51
Signal transduction_Activin A signaling regulation	6.82E–08	12/33
Transcription_Role of heterochromatin protein 1 (HP1) family in transcriptional silencing	7.00E–08	10/22
Development_Alpha-1 adrenergic receptors signaling via cAMP	2.09E–07	9/19

**Note: GeneGo pathway analysis of putative targeted genes of synergistically regulated miRNAs at Progression stage. The 10 most significantly enriched pathways were listed in table1. The detailed enriched pathways were shown in Table S5.**

Notably, the Hdac-related pathway was the second most significantly enriched one. Although studies reported that Hdac is involved in ESC differentiation [30], [31], whether Hdac is indeed involved in Wnt3a and Activin A induced DE differentiation is not known. At DE maturation stage, we also predicted target genes for these candidate miRNAs. The results indicated that many pathways which were significantly enriched in DE progression were also significantly enriched during DE maturation (Table 2). Besides the Hdac-related pathway, Sin3 and NuRD pathways were also significantly enriched. Sin3 and NuRD are two members that form complexes with Hdacs to regulate gene expression. We further used Miranda to predict these miRNA target genes and further used the predicted genes in the pathway analysis. Hdac and related pathways could be also significantly enriched (data not shown). Together, the data suggest that Hdac might play an important role in DE formation.

**Table 2:** The 10 most significantly enriched pathways at DE maturation stage.

Name	pValue	Network objects
Transcription_Sin3 and NuRD in transcription regulation	6.79E–11	16/38
Development_Thrombopoietin-regulated cell processes	1.25E–08	15/45
Cell cycle_ESR1 regulation of G1/S transition	1.22E–07	12/33
Stem cells_Self-renewal of adult neural stem cells	2.56E–07	14/48
Transcription_Ligand-dependent activation of the ESR1/SP pathway	3.76E–07	11/30
Cardiac Hypertrophy_NF-AT signaling in Cardiac Hypertrophy	4.70E–07	16/65
Development_PDGF signaling via STATs and NF-kB	8.00E–07	11/32
Signal transduction_Activin A signaling regulation	1.14E–06	11/33
Development_Role of HDAC and calcium/calmodulin-dependent kinase (CaMK) in control of skeletal myogenesis	1.25E–06	14/54
Development_HGF signaling pathway	1.35E–06	13/47

**Note: GeneGo pathway analysis of putative targeted genes of synergistically regulated miRNAs at maturation stage. The 10 most significantly enriched pathways were listed in** **table 2** **. The detailed enriched pathways were shown in Table S6.**

### Regulation of histone acetylation during DE differentiation

**Figure 6:**
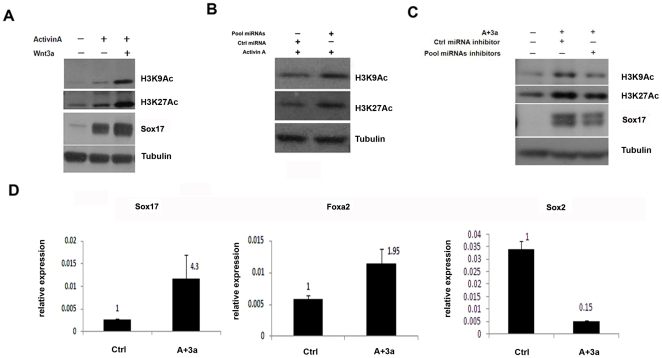
Involvement of histone acetylation in DE differentiation. A. Wnt3a increases H3K9/K27 acetylation level during DE differentiation. Mouse ESCs were treated with Activin A and Wnt3a for 5 days and subjected to western blot analysis on histone H3 acetylation using antibodies against H3K9ac and H3K27ac. Sox17 was examined to confirm DE differentiation. Tubulin was used as the loading control. B. Western blot analysis on histone H3 acetylation using antibodies against H3K9ac and H3K27ac in pooled miRNA over-expressed DE samples. Tubulin was used as the loading control. Please note that over-expression of a subset of miRNAs increases H3K9/K27 acetylation level. C. Western blot analysis on Sox 17 and histone H3 acetylation using antibodies against H3K9ac and H3K27ac in pooled miRNA inhibitors over-expressed DE samples. Tubulin was used as the loading control. D. the H3K9 acetylation level of Sox17, Foxa2, and Sox2 promoter regions was quantified by ChIP-qPCR. The data was normalized to input; fold change values were labeled as indicated. Data are expressed as mean percentage ± SD. A: Activin A; 3a:Wnt3a.

In order to confirm the roles of Hdac in DE formation, we checked histone H3 acetylation level during DE differentiation. Treating the cells with Activin A resulted in increased histone acetylation as manifested by western blot analyses on both histone H3K9 and H3K27 acetylation (Figure 6A). Moreover, the increase of histone acetylation is more significant when the cells were co-treated with Activin A and Wnt3a. There is no significant change of total histone (data not shown). Since the involvement of Hdac in DE differentiation was identified through pathway analysis on miRNA expression, we next asked if Wnt3a and Activin A induced histone acetylation could be mediated through miRNA. We thus over expressed the pooled miRNA as described above and examined the histone acetylation level by western blot analysis. The data demonstrated that forced expression of these miRNA also partially increased histone acetylation (Figure 6B). To further confirm the roles of these regulatory miRNAs, we tried to inhibit the expression of the miRNAs by using synthetic miRNA inhibitors during Activin A and Wnt3a induced DE differentiation. When the cells were transfected with the pooled miRNA inhibitors against the 6 regulatory miRNAs that have been tested in the over-expression experiment, miRNA inhibitory efficiency was further examined by using RT-qPCR (Figure S5). The result shown that the increased histone acetylation level and DE marker expression induced by Activin A and Wnt3a were attenuated (Figure 6C). These data indicated that Wnt3a and Activin A induced histone acetylation and DE differentiation could be at least partially mediated through miRNAs.

### Histone acetylation of the promoter regions of Sox17 and Foxa2

We next asked if the promoters of DE-specific transcription factors could be direct targets of histone acetylation regulation. We first performed chromatin IP PCR (ChIP_PCR) experiments to assess the level of histone acetylation at the promoter regions of Sox17 and Foxa2. The pluripotent marker Sox2 was also assessed as a control. We surveyed 1 k, 2 k, and 50 k upstream of the promoter regions of Sox17 and Foxa2. The results demonstrated that acetylated H3K9 was indeed recruited to the promoter regions of Sox17 (1 k and 2 k regions of the promoter) and Foxa2 (1 k, 2 k, and 50 k regions of the promoter) in both differentiated and undifferentiated cells. Interestingly, H3K9ac level at 2 k region of Sox17 and Foxa2 promoters were significantly higher in Activin A and Wnt3a treated cells than that of the control cells by ChIP-quantitative PCR analysis (Figure 6D). As the control, the H3K9ac level at Sox2 promoter was significantly decreased upon Activin A and Wnt3a treatment which is opposed to that of Sox17 and Foxa2. Since H3K9ac is an active histone marker, our data suggested that the expression of key DE transcription factors like Sox17 and Foxa2 is subject to transcription activation through histone acetylation during Activin A and Wnt3a induced DE formation.

### Inhibition of Hdac activity promotes Activin A-induced DE differentiation

In order to further explore which Hdac(s) might be involved in regulating Activin A and wnt3a induced DE differentiation, we checked which Hdac(s) could be targeted by the synergistic miRNAs by using alignment analysis using miRNA consensus sequences. The results showed four consensus binding sites for the miRNAs (mi181C, 338-5p, 196a/b) in the Hdac9 3′ UTR region. There is also one consensus miRNA (338-5p) binding sequence for Hdac7 at the 3′ UTR after poly A signal region (Figure S6). We next checked these Hdac expression in miRNA pooled over-expressed differentiating cells. We found that Hdac9 not Hdac7 could be significantly down-regulated, suggesting that Hdac9 might be involved in mouse DE differentiation (Figure S7).

**Figure 7:**
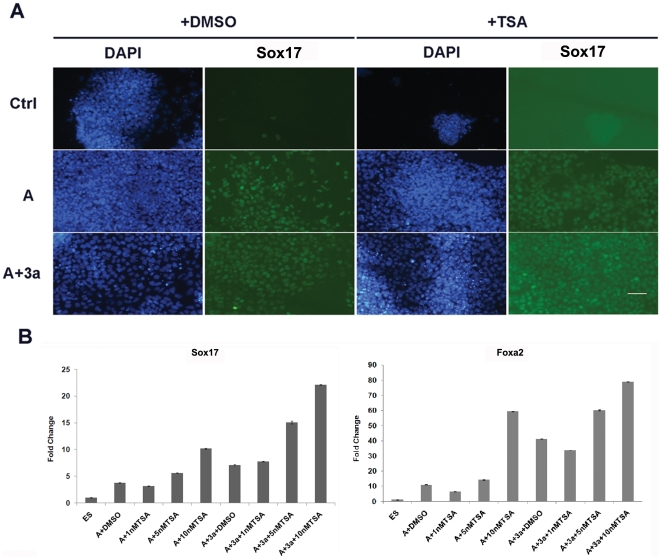
Inhibiting of histone de-acetylation by TSA. A. IF staining of Sox17 in control and 10 nM TSA treated differentiated samples. TSA was added at the final concentration of 10 nM to the differentiation medium for five days. DMSO was added as the negative control. Please note that addition of TSA greatly increases the DE differentiation efficiency. Scale bar: 50 uM. B. Sox17 and Foxa2 expression in cells treated with different doses of TSA was examined by RT-qPCR. Mouse ESCs were treated the same as above, TSA was added at the final concentration ranging from 1 nm to 10 nM to the differentiation medium for five days as indicated. Please note that TSA dose dependently increases the DE marker expression. Data are expressed as mean fold change ± SD. A: Activin A; 3a:Wnt3a.

We further treated the cells with the Hdac inhibitor trichostatin A (TSA) in our differentiation experiments. RT-qPCR and IF staining were applied to assess DE differentiation 5 days after treatment. The results revealed that TSA could significantly increase the efficiency of Activin A induced DE differentiation (Figure 7A and 7B), notably, under Activin A, Wnt3a and TSA co-treatment, almost all of the cells are Sox17 positive. Using other Hdac inhibitors, we obtained similar results (data not shown). These data indicated that inhibiting Hdac activity does promote DE differentiation from mouse ESCs.

## Discussion

In order to understand the molecular mechanisms underlying DE differentiation, we analyzed genome wide miRNA expression in Activin A and Wnt3a-induced DE formation from mouse ESCs. We applied various informatics approaches including SOM-based clustering analysis to establish the miRNA expression fingerprint of DE differentiation. We identified a subset of miRNAs whose expression was synergistically regulated by Wnt3a and Activin A. Forced expression of those synergistically regulated miRNAs partially mimics the roles of Wnt3a, suggesting that they are likely to play regulatory roles guiding DE differentiation. Pathway analyses revealed that several key pathways maybe regulated by miRNA during DE differentiation. Among them includes the Hdac pathway. Consistent with the miRNA profiling results, Activin A and Wnt3a treatment increases the level of histone H3 acetylation, while inhibition of Hdac greatly enhances the DE differentiation efficiency. ChIP analysis further revealed that the key DE transcription factors are subject to transcription activation through histone acetylation. Together, the present miRNA profiling studies revealed novel epigenetic insights into DE differentiation.

Wnt signaling stimulates numerous intracellular signal transduction cascades to regulate stem cell self-renewal and differentiation. *In vivo*, Wnt signaling plays an important role during gastrulation [7], [8]. Wnt-mediated Brachyury expression is also important for the migration of precursor cells through the anterior region of the primitive streak (PS). The subsequent specification of the anterior region of the PS to mesoderm or endoderm is thought to depend on the duration and magnitude of Nodal signaling [9], [32], [33]. Following its commitment, the DE cells line the ventral region of the developing embryo and are patterned by adjacent mesenchyme. *In vitro*, inhibiting Wnt signaling can inhibit the differentiation of mouse hepatic progenitor cells [34]. Our data indicated that Wnt3a activates beta-catenin activity in mouse ESCs during DE differentiation (not shown), and further affects miRNA expression to facilitate Activin A-induced DE formation. Particularly, when DE differentiation proceeds, we found that an increased number of miRNAs were differentially regulated by Wnt3a and Activin A co-treatment, suggesting that Wnt3a might play more important roles in the late stages of DE formation. The results are consistent with previous findings that canonical Wnt signaling is required only during late stages of Activin A-induced development of Sox17-expressing endoderm cells [35]. Wnt3a treatment alone does not significantly affect miRNA expression. We noticed that there were many miRNAs, such as mir-340-3p, mir-340-5p, mir-1896, mir-546, and mir-301b, whose expression were not significantly affected by Activin A or Wnt3a alone at progression or maturation stages, but regulated by Activin A and Wnt3a co-treatment. Interestingly, all of these miRNAs were synergistically down-regulated in Activin A and Wnt3a treated samples. The functions of these synergistically down-regulated miRNAs in DE differentiation warrant further detailed characterization. Moreover, although the synergistically regulated miRNAs upon Activin A and Wnt3a treatment could be validated by RT-qPCR analyses, we found the expression pattern of primary miRNA do not have this pattern (data not shown), which suggested that Activin A and Wnt 3a might mainly affect miRNA processing but not their expression per se.

Our functional analyses revealed that many putative pathways were significantly enriched by synergistically regulated miRNAs. Although, synergistically regulated miRNAs were not completely over-lapped between DE progression and maturation stage, most of the pathways they supposedly regulate were over-lapped, suggesting that Wnt3a plays a consistent role in DE progression and maturation, but through different miRNAs. Among the putative pathways, TGF-beta, Notch and FGF pathways, have been reported to be involved in DE formation *in vitro* and *in vivo* [36]–[38], suggesting that Wnt3a may target multiple pathways to promote DE differentiation from mouse ESCs.

Importantly, our pathway analysis on the synergistically regulated miRNAs revealed the possible involvement of Hdac in DE differentiation. Modification of chromatin by epigenetic regulatory mechanisms, including histone acetylation, deacetylation and methylation, is considered as an important mechanism for stem cell pluripotency and differentiation. Hdacs have been reported to play important roles governing stem cell aging and differentiation by regulating genes and miRNAs expression. Moreover, recent studies revealed that Hdac forms a complex with beta-catenin and regulates beta-catenin localization [39], [40], suggesting cross-talk between Hdacs and Wnt downstream pathways. We examined publically available microarray data and found that the expression level of Sox17 is significantly up-regulated in Hdac1 knockout ESCs when compared to wild type cells [41]. Two Hdac library derived small molecules could significantly induce DE differentiation from mouse and human ESCs [25]. There are no direct evidences for Hdac involvement in Wnt3a-induced DE differentiation. In this report, we demonstrated that Wnt3a increases histone H3K9/K27 acetylation levels in Activin A-induced DE, which could be partially mimicked by forced expression of a subset of miRNAs. Inhibiting Hdac activity promoted Activin A-induced DE differentiation. Moreover, Sox17 and Foxa2 promoters are indeed acetylated, indicating a molecular cross talk among Wnt3a, Hdac activity, gene expression and DE differentiation. However, how Wnt3a regulates Hdac activity and how Hdacs affect DE differentiation and which Hdac(s) is mainly involved in this process are key questions that need further investigation.

In recent years, researches suggested that Hdac inhibition accelerates the early events of stem cell differentiation [42]. Inhibiting Hdac activity could promote osteogenic differentiation, neural cells differentiation, and myocardial differentiation, respectively [30], [43], [44]. We noticed that treatment of TSA alone also increased other germ line layer marker expression such as Cdx2, etc (data not shown). Together with previous studies showing that Hdacs are involved in neural cell differentiation [43], it is likely that inhibiting Hdacs might open chromatin and render cells the flexibility allowing stem cell differentiation. The roles of Hdac on DE differentiation maybe only permissive but not instructive and it might not be lineage-specific either. A very recent report suggested that pre-treatment of TSA increases DE differentiation from human ES cells, which also supports the current hypothesis [45].

Mouse embryonic development is a good model system to understand human embryo and organ development. They share some common mechanisms. We also examined whether miRNAs involved in mouse DE differentiation also regulate human DE differentiation from human ESCs. We compared our results with previously published miRNAs data about human DE differentiation [18], [19], and found that there are many miRNAs over-lapping between mouse and human data, such as mir-196a/b, let-7e and so on, suggesting the molecular conservativeness between human and mouse DE differentiation in terms of miRNA expression.

In summary, miRNAs are emerging as key regulators for ESCs self-renewal and differentiation. Genome wide miRNA expression profiling could reveal novel molecular insights into DE differentiation. The involvement of histone acetylation in DE differentiation uncovered by miRNA profiling and pathway analyses established a regulatory cascade from extracellular cytokine treatment to miRNA expression to histone modification in cell nuclei. Our results also support a new venue to increase DE differentiation for regenerative medicine through inhibition of Hdac activity.

## Supporting Information

Figure S1 **Hierarchal clustering (A) and PCA (B) analyses of raw data of miRNA profiling.** The raw data was normalized in GeneSpring, subsequent clustering was performed using R, and PCA using GeneSpring.
(TIF)

Figure S2 **Known DE specific miRNA expression during Activin A and Wnt3a induced DE differentiation.** Mouse ESCs were treated with 100 ng/ml Activin A, 50 ng/ml Wnt3a, or 100 ng/ml Activin A plus 50 ng/ml Wnt3a as indicated. The differentiated cells samples were collected at day 5 of differentiation for miRNA expression analysis using Taqman RT-qPCR. The expression of two miRNAs identified in human DE differentiation, mir-125a-5p and mir-let-7e, were analyzed as presented. Data are expressed as mean fold change ± SD. A: Activin A; 3a:Wnt3a.
(TIF)

Figure S3 **RT-qPCR examines selected miRNAs expression in differentiated mouse ESCs.** Mouse ESCs were treated with 100 ng/ml Activin A, 50 ng/ml Wnt3a, or 100 ng/ml Activin A plus 50 ng/ml Wnt3a as indicated. The differentiated cell samples were collected at day 5 of differentiation for miRNA expression analysis using Taqman RT-qPCR. Data are expressed as mean fold change ± SD. A:Activin A; 3a:Wnt3a.
(TIF)

Figure S4 **RT-qPCR examines miRNAs expression in pooled miRNA over-expressed differentiated mouse ESCs.** Mouse ESCs were treated with100 ng/ml Activin A for three days, then transfected with pooled miRNAs to allow further differentiation for 2 more days. Cel-miR-67 was used as a negative control. The differentiated cell samples were collected at day 5 of differentiation for miRNA expression analysis using Taqman RT-qPCR. Data are expressed as mean fold change ± SD. A:Activin A; 3a:Wnt3a.
(TIF)

Figure S5 **RT-qPCR examines miRNAs expression in pooled miRNA inhibitor over-expressed differentiated mouse ESCs.** Mouse ESCs were treated with100 ng/ml Activin A and 50 ng/ml Wnt3a for three days, then transfected with pooled miRNAs inhibitors to allow further differentiation for 2 more days. Cel-miR-67 inhibitor was used as a negative control. The differentiated cell samples were collected at day 5 of differentiation for miRNA expression analysis using Taqman RT-qPCR. Data are expressed as mean fold change ± SD. A: Activin A; 3a:Wnt3a.
(TIF)

Figure S6 **Consensus miRNA binding sites in the 3′ UTR region of Hdac7 and Hdac9.** A. The consensus miRNA binding sites in the 3′ UTR region of Hdac7; B. The consensus binding miRNA sites in the 3′ UTR region of Hdac9. Red: mir-338-5p binding site; Yellow: mir-181c binding site; Blue: mir-196 a/mir-196b binding site.
(TIF)

Figure S7 **RT-qPCR examination of Hdac expression in pooled miRNA over-expressed differentiated mouse ESCs.** Mouse ESCs were treated with100 ng/ml Activin A for three days, then transfected with pooled miRNAs to allow further differentiation for 2 more days. Cel-miR-67 was used as a negative control. The differentiated cell samples were collected at day 5 of differentiation for HDACs expression analysis using RT-qPCR. Data are expressed as mean fold change ± SD. A: Activin A; 3a:Wnt3a.
(TIF)

Methods S1 **miRNA over-expression protocol.**
(DOC)

Table S1 **Primers sequences.** Listed are the primer sequences for qPCR analyses and the miRNA Taqman assay IDs. The PCR primers were synthesized from Invitrogen (Carlsbad, USA). All miRNA Taqman assays were purchased from ABI (Foster City, USA).
(DOC)

Table S2 **Differentially expressed genes in all samples.** Mouse ESCs were treated with 100 ng/ml Activin A, 50 ng/ml Wnt3a, or 100 ng/ml plus 50 ng/ml Wnt3a, and the samples were collected and profiled at 1 day, 3 days, and 5 days after treatment. The differentially expressed miRNAs were identified by using ANOVA test under FDR <0.05. Total of 386 differentially expressed miRNAs were identified.
(XLS)

Table S3 **Differentially expressed miRNAs at day3 samples.** Identification of differentially expressed miRNAs was described in the methods section. 90 differentially expressed miRNAs under the cutoff as FDR <0.05 and fold>2 or <0.5 were identified from the 3 day samples.
(XLS)

Table S4 **Differentially expressed miRNAs at day5 samples.** Identification of differentially expressed miRNAs was described in the methods section. 118 differentially expressed miRNAs under the cutoff as FDR <0.05 and fold>2 or <0.5 were identified from the 5 day samples.
(XLS)

Table S5 **Enriched pathways of synergistically regulated miRNAs at DE progression stages.** GeneGo pathway analysis of putative targeted genes was performed on the synergistically regulated miRNAs at the DE progression stage. 300 pathways were significantly enriched.
(DOC)

Table S6 **Enriched pathways of synergistically regulated miRNAs at DE maturation stages.** GeneGo pathway analysis of putative targeted genes was performed on the synergistically regulated miRNAs at the DE maturation stage. 252 pathways were significantly enriched.
(DOC)
