# Supplementary figures and images for: Involvement of Histone Acetylation of Sox17 and Foxa2 Promoters during Mouse Definitive Endoderm Differentiation Revealed by MicroRNA Profiling
Source: PLoS One. 2011 Nov 23;6(11):e27965. doi: 10.1371/journal.pone.0027965 (PMC3223193; doi:10.1371/journal.pone.0027965)

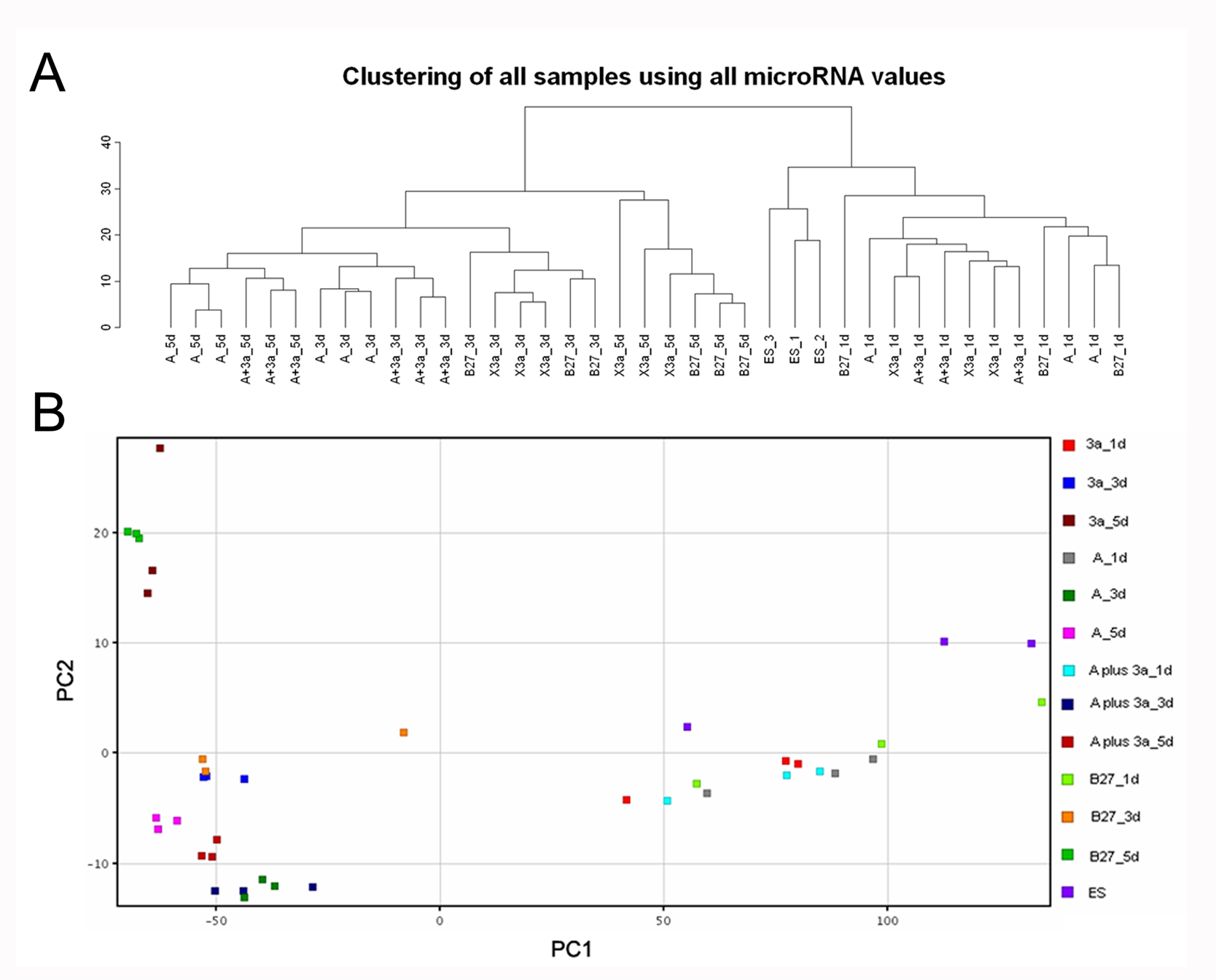

Supplement: Figure S1 — Hierarchal clustering (A) and PCA (B) analyses of raw data of miRNA profiling. The raw data was normalized in GeneSpring, subsequent clustering was performed using R, and PCA using GeneSpring. (TIF) [file pone.0027965.s001.tif]

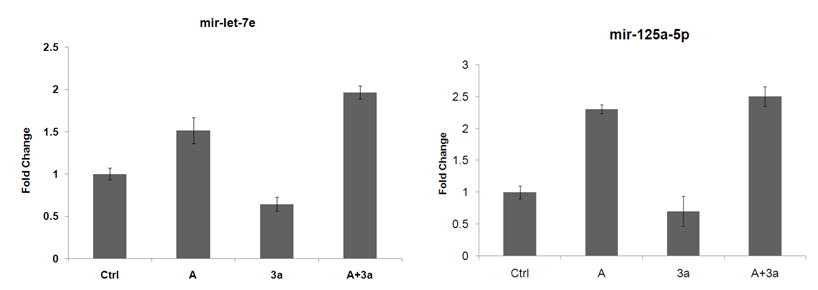

Supplement: Figure S2 — Known DE specific miRNA expression during Activin A and Wnt3a induced DE differentiation. Mouse ESCs were treated with 100 ng/ml Activin A, 50 ng/ml Wnt3a, or 100 ng/ml Activin A plus 50 ng/ml Wnt3a as indicated. The differentiated cells samples were collected at day 5 of differentiation for miRNA expression analysis using Taqman RT-qPCR. The expression of two miRNAs identified in human DE differentiation, mir-125a-5p and mir-let-7e, were analyzed as presented. Data are expressed as mean fold change ± SD. A: Activin A; 3a:Wnt3a. (TIF) [file pone.0027965.s002.tif]

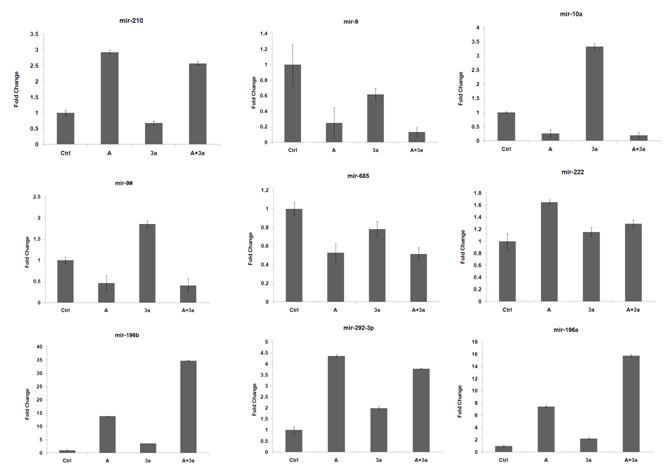

Supplement: Figure S3 — RT-qPCR examines selected miRNAs expression in differentiated mouse ESCs. Mouse ESCs were treated with 100 ng/ml Activin A, 50 ng/ml Wnt3a, or 100 ng/ml Activin A plus 50 ng/ml Wnt3a as indicated. The differentiated cell samples were collected at day 5 of differentiation for miRNA expression analysis using Taqman RT-qPCR. Data are expressed as mean fold change ± SD. A:Activin A; 3a:Wnt3a. (TIF) [file pone.0027965.s003.tif]

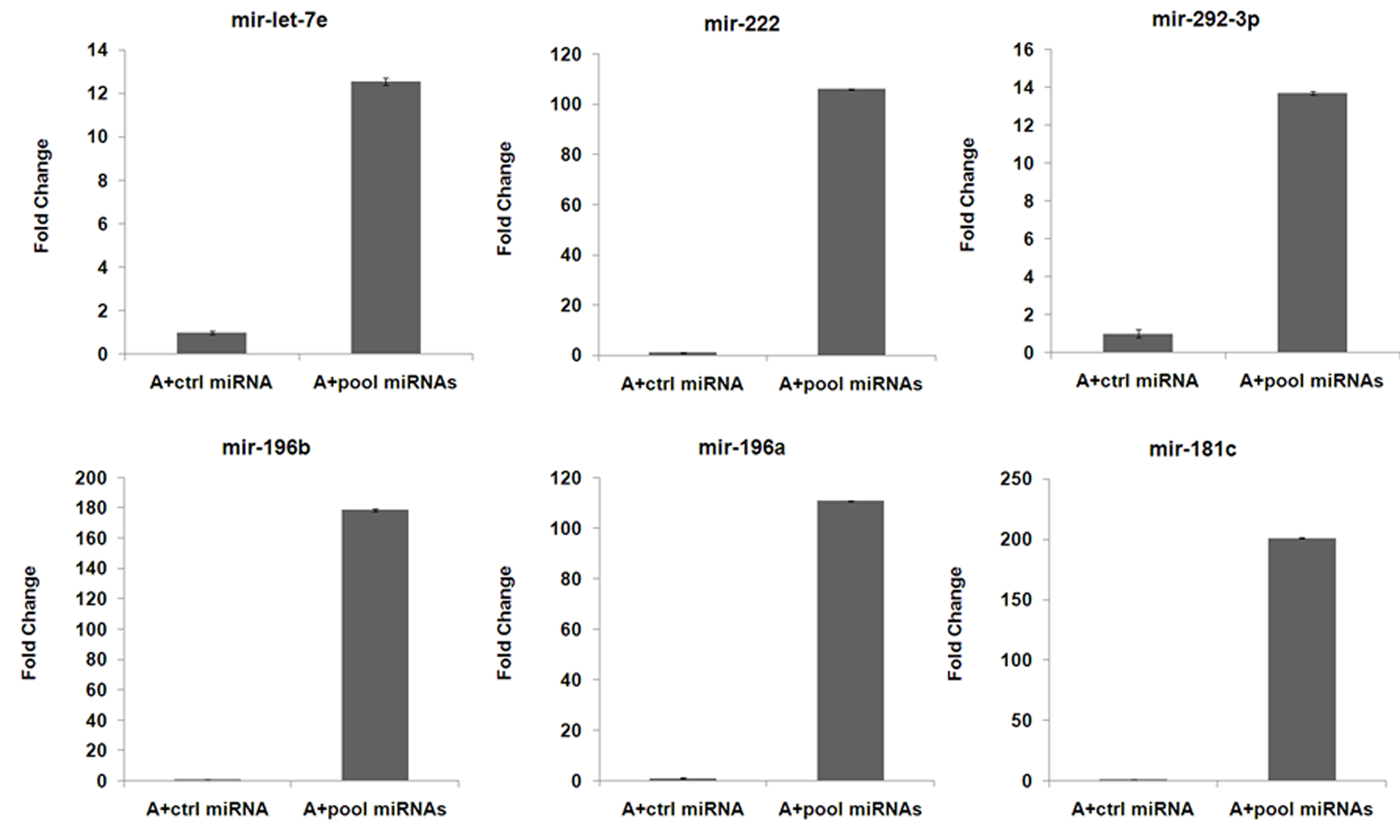

Supplement: Figure S4 — RT-qPCR examines miRNAs expression in pooled miRNA over-expressed differentiated mouse ESCs. Mouse ESCs were treated with100 ng/ml Activin A for three days, then transfected with pooled miRNAs to allow further differentiation for 2 more days. Cel-miR-67 was used as a negative control. The differentiated cell samples were collected at day 5 of differentiation for miRNA expression analysis using Taqman RT-qPCR. Data are expressed as mean fold change ± SD. A:Activin A; 3a:Wnt3a. (TIF) [file pone.0027965.s004.tif]

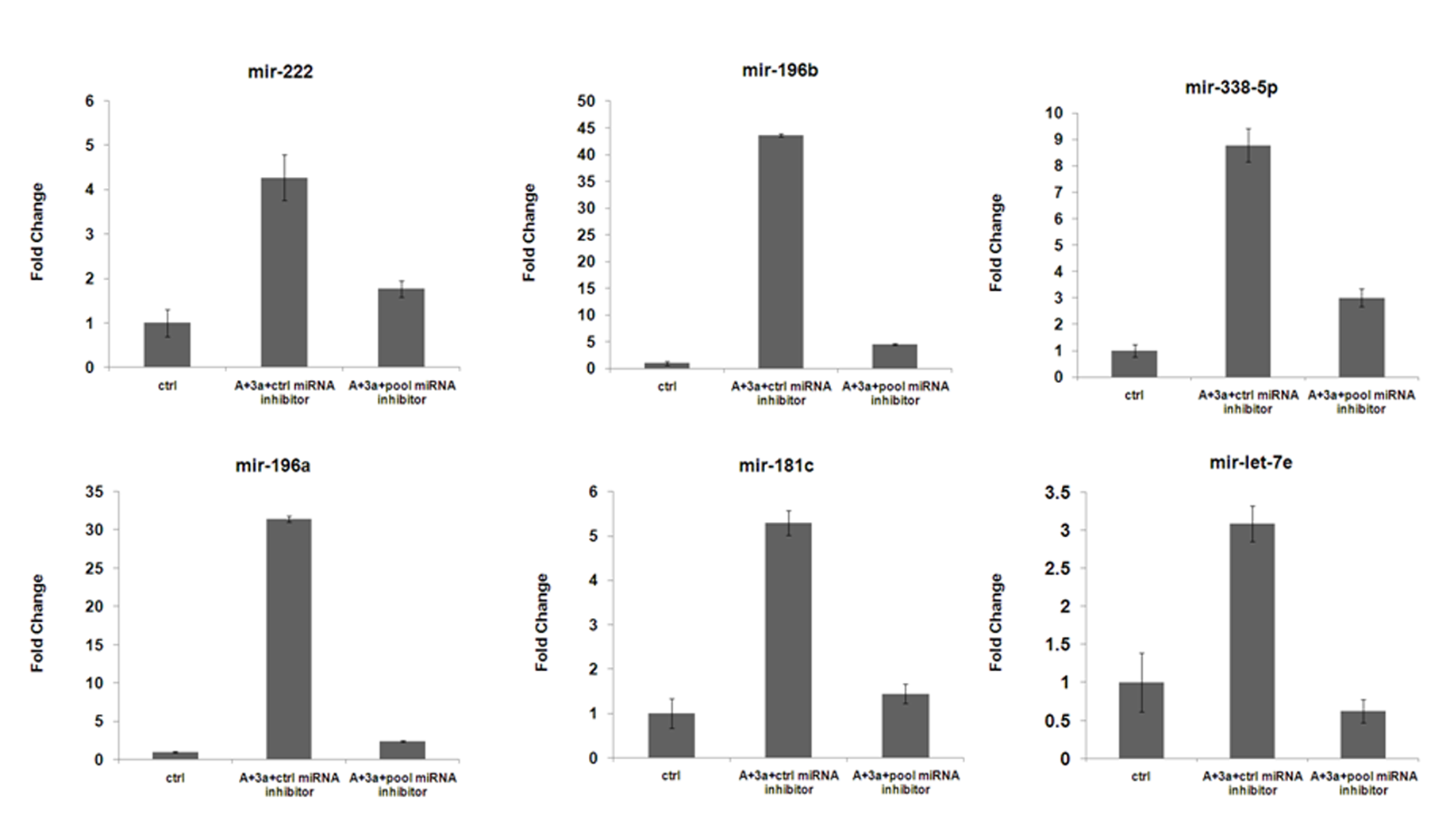

Supplement: Figure S5 — RT-qPCR examines miRNAs expression in pooled miRNA inhibitor over-expressed differentiated mouse ESCs. Mouse ESCs were treated with100 ng/ml Activin A and 50 ng/ml Wnt3a for three days, then transfected with pooled miRNAs inhibitors to allow further differentiation for 2 more days. Cel-miR-67 inhibitor was used as a negative control. The differentiated cell samples were collected at day 5 of differentiation for miRNA expression analysis using Taqman RT-qPCR. Data are expressed as mean fold change ± SD. A: Activin A; 3a:Wnt3a. (TIF) [file pone.0027965.s005.tif]

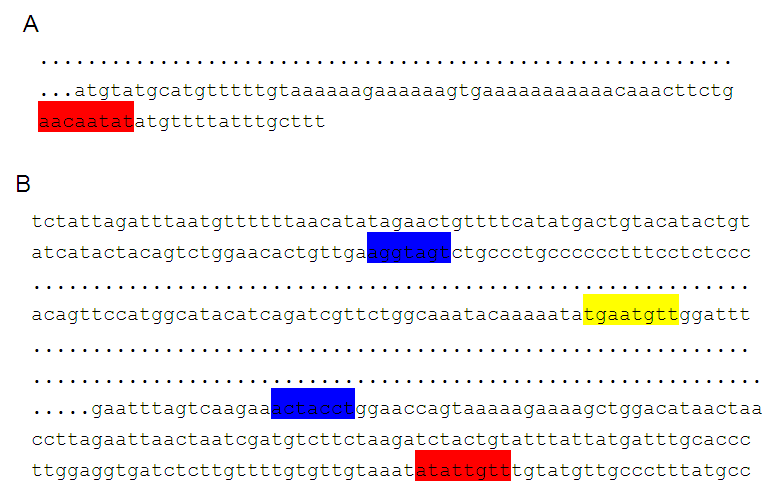

Supplement: Figure S6 — Consensus miRNA binding sites in the 3′ UTR region of Hdac7 and Hdac9. A. The consensus miRNA binding sites in the 3′ UTR region of Hdac7; B. The consensus binding miRNA sites in the 3′ UTR region of Hdac9. Red: mir-338-5p binding site; Yellow: mir-181c binding site; Blue: mir-196 a/mir-196b binding site. (TIF) [file pone.0027965.s006.tif]

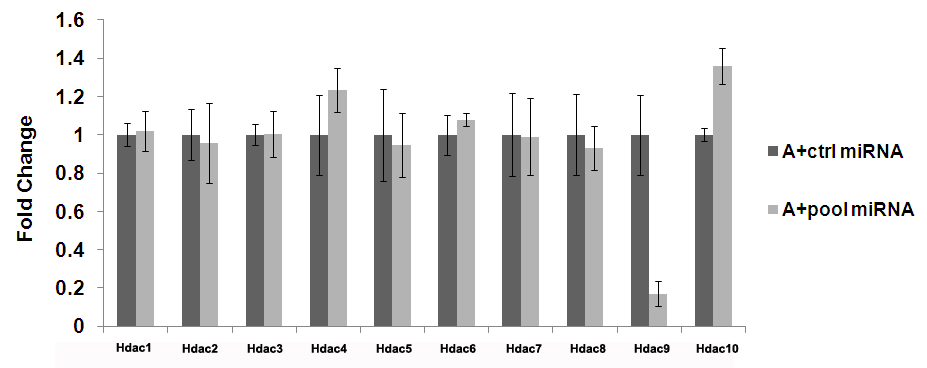

Supplement: Figure S7 — RT-qPCR examination of Hdac expression in pooled miRNA over-expressed differentiated mouse ESCs. Mouse ESCs were treated with100 ng/ml Activin A for three days, then transfected with pooled miRNAs to allow further differentiation for 2 more days. Cel-miR-67 was used as a negative control. The differentiated cell samples were collected at day 5 of differentiation for HDACs expression analysis using RT-qPCR. Data are expressed as mean fold change ± SD. A: Activin A; 3a:Wnt3a. (TIF) [file pone.0027965.s007.tif]
