# Supplementary material for: Involvement of Histone Acetylation of Sox17 and Foxa2 Promoters during Mouse Definitive Endoderm Differentiation Revealed by MicroRNA Profiling
Source: PLoS One. 2011 Nov 23;6(11):e27965. doi: 10.1371/journal.pone.0027965 (PMC3223193; doi:10.1371/journal.pone.0027965)
Supplement: Methods S1 — miRNA over-expression protocol. (DOC) [file pone.0027965.s008.doc]

**miRNA over-expression protocol**

Day 0:

1. 6-well plate was coated with collagen at the final concentration of 0.05mg/ml (diluted with 0.02N acetic acid)

2. mES cells were split and incubated for 1h to remove feeder cells

3. 80000 cells /per well were plated to the collagen coated 6-well plate in mES culture medium.

Day 1:

1. The culture medium was changed to 2ml/well differentiation medium (PRIM1640+1XB27+1Xsodium byruate+100ng/ml Activin A)

Day 2:

1. Medium was changed the same as above

Day 3:

1. The cultures were fed with fresh differentiation medium (PRIM1640+1XB27+1Xsodium byruate+100ng/ml Activin A)

2. Six synthetic mature miRNAs, mir-181c/338-5p/222/196a/196b/let-7e, were pooled together equivalently. The final concentration for pooled miRNAs and control miRNA(Cel-67) is 20uM.

3. Cells were transfect with 10ul miRNAs using 5 ul RNAiMAX reagent according to manufacture’s protocol

Day 4:

1. Medium was refreshed with 2ml differentiation medium the same as above

Day 5:

1. The cultures were replenished with the differentiation medium and re-transfected with the pooled miRNA and the control miRNA the same as the day 3 initial transfection

Day6:

1. The cells were washed once with PBS and lysed in RNA lysis buffer (Qiagen) for RT-qPCR or in RIPA buffer for western-blot analyses
