# Supplementary material for: Involvement of Histone Acetylation of Sox17 and Foxa2 Promoters during Mouse Definitive Endoderm Differentiation Revealed by MicroRNA Profiling
Source: PLoS One. 2011 Nov 23;6(11):e27965. doi: 10.1371/journal.pone.0027965 (PMC3223193; doi:10.1371/journal.pone.0027965)
Supplement: Table S1 — Primers sequences. Listed are the primer sequences for qPCR analyses and the miRNA Taqman assay IDs. The PCR primers were synthesized from Invitrogen (Carlsbad, USA). All miRNA Taqman assays were purchased from ABI (Foster City, USA). (DOC) [file pone.0027965.s009.doc]

**Table S1. Primers sequences**

**Primers for real-time PCR**

| Gene | Primer sequence |
| --- | --- |
| Sox17_F | gctaggcaagtcttggaagg |
| Sox17_R | cttgtagttggggtggtcct |
| Foxa2_F | tggctgcagacacttcctac |
| Foxa2_R | aagctctcccaaagtctcca |
| mGapdh_F | aacgggaagcccatcacc |
| mGapdh_R | cagccttggcagcaccag |
| Ck7_F | caggatgtggtggaggactt |
| Ck7_R | ttgctcatgtaggcagcatc |
| Ck18_F | cgaggcactcaaggaagaac |
| Ck18_R | gctgaggtcctgagatttgg |
| Ck19_F | ggtcagtgtggaggtggatt |
| Ck19_R | cctcaatccgagcaaggtag |
| Hnf4_F | agaggttctgtcccagcaga |
| Hnf4_R | atgtacttggcccactcgac |
| Tat_F | agccattgtggacaacatga |
| Tat_R | cttcatggcttgggtcactt |
| G6p_F | atgactttgggatccagtcg |
| G6p_R | tggaaccagatgggaaagag |
| Hdac1_F | tgctgtgaactacccactgc |
| Hdac1_R | cactgcactaggctggaaca |
| Hdac2_F | tgctgtcaattttcccatga |
| Hdac2_R | gcgctaggctggtacatctc |
| Hdac3_F | tgcttcaatctcagcattcg |
| Hdac3_R | ggacagtgtagccaccacct |
| Hdac4_F | aaatgagtttgccccagatg |
| Hdac4_R | acccaaaacatttggcagag |
| Hdac5_F | aggaggaagaggaggactgc |
| Hdac5_R | gtacacctggaggggctgta |
| Hdac6_F | gcaacctctgggacaatcat |
| Hdac6_R | gtggcaggtaaggagctcag |
| Hdac7_F | gaagctggctgaagtgatcc |
| Hdac7_R | ccaagggctcaagagttctg |
| Hdac8_F | tgccctgcataaacaaatga |
| Hdac8_R | ctggatggtcctcatcacct |
| Hdac9_F | cttactcaggcggaaggatg |
| Hdac9_R | gcctcattttcggtcacatt |
| Hdac10_F | cagaggaagagttgggcttg |
| Hdac10_R | ggtgtccgggtgaaagtaga |

Probe ID for Taqman miRNA

| Assay ID miRNA name |
| --- |
| Assay ID-002215 mmu-miR-196b |
| Assay ID-000495 mmu-miR-196a |
| Assay ID-002406 mmu-let-7e |
| Assay ID-000583 mmu-miR-9 |
| Assay ID-002231 mmu-miR-9* |
| Assay ID-001670 mmu-miR-685 |
| Assay ID-002276 mmu-miR-222 |
| Assay ID-002593 mmu-miR-292-3p  Assay ID_000482 mmu-miR -181c |
| Assay ID-000387 mmu-miR-10a |
| Assay ID-000512 mmu-miR-210 |
| Assay ID-002198 mmu-mir-125a-5p |
| Assay ID-001973 U6 snRNA |

**Primers of real-time PCR For CHIP**

| Gene Name | Primer sequence |
| --- | --- |
| mSOX17F_2K | cctttcctcaagcactcagg |
| mSOX17R_2K | aaacctgccctagatgctca |
| mFOXA2F_2K | cccagtgtctcaggaatggt |
| mFOXA2R_2K | tggaggctcacaaaataccc |
| mSox2_F_2k | cagaaacaatggcacaccac |
| mSox2_R_2k | caagacgacagctcctttcc |
