# Supplementary material for: Involvement of Histone Acetylation of Sox17 and Foxa2 Promoters during Mouse Definitive Endoderm Differentiation Revealed by MicroRNA Profiling
Source: PLoS One. 2011 Nov 23;6(11):e27965. doi: 10.1371/journal.pone.0027965 (PMC3223193; doi:10.1371/journal.pone.0027965)
Supplement: Table S5 — Enriched pathways of synergistically regulated miRNAs at DE progression stages. GeneGo pathway analysis of putative targeted genes was performed on the synergistically regulated miRNAs at the DE progression stage. 300 pathways were significantly enriched. (DOC) [file pone.0027965.s013.doc]

Table S5.enriched pathways of synergistically regulated miRNAs at progression stages.

| Name | pValue | Network objects |
| --- | --- | --- |
| Development_TGF-beta receptor signaling | 3.77E-12 | 19/50 |
| Development_Role of HDAC and calcium/calmodulin-dependent kinase (CaMK) in control of skeletal myogenesis | 1.86E-11 | 19/54 |
| Cytoskeleton remodeling_TGF, WNT and cytoskeletal remodeling | 2.08E-11 | 27/111 |
| Cell adhesion_Ephrins signaling | 6.09E-09 | 15/45 |
| Development_Slit-Robo signaling | 1.93E-08 | 12/30 |
| Stem cells_Early embryonal hypaxial myogenesis | 3.10E-08 | 13/37 |
| Development_IGF-1 receptor signaling | 4.17E-08 | 15/51 |
| Signal transduction_Activin A signaling regulation | 6.82E-08 | 12/33 |
| Transcription_Role of heterochromatin protein 1 (HP1) family in transcriptional silencing | 7.00E-08 | 10/22 |
| Development_Alpha-1 adrenergic receptors signaling via cAMP | 2.09E-07 | 9/19 |
| Signal transduction_PKA signaling | 3.07E-07 | 14/51 |
| Neurophysiological process_Receptor-mediated axon growth repulsion | 4.26E-07 | 13/45 |
| Immune response_IL-2 activation and signaling pathway | 1.26E-06 | 13/49 |
| Stem cells_Astrocyte differentiation from adult stem cells | 1.32E-06 | 11/35 |
| Cardiac Hypertrophy_NF-AT signaling in Cardiac Hypertrophy | 1.34E-06 | 15/65 |
| Immune response_Function of MEF2 in T lymphocytes | 1.61E-06 | 13/50 |
| Transport_RAN regulation pathway | 1.87E-06 | 8/18 |
| Transcription_Ligand-dependent activation of the ESR1/SP pathway | 2.20E-06 | 10/30 |
| Development_Glucocorticoid receptor signaling | 2.31E-06 | 9/24 |
| Transcription_CREB pathway | 2.35E-06 | 12/44 |
| Immune response_Gastrin in inflammatory response | 3.01E-06 | 15/69 |
| Cytoskeleton remodeling_Reverse signaling by ephrin B | 3.09E-06 | 10/31 |
| Transcription_Sin3 and NuRD in transcription regulation | 3.27E-06 | 11/38 |
| Signal transduction_cAMP signaling | 3.27E-06 | 11/38 |
| Stem cells_Differentiation of white adipocytes | 3.30E-06 | 13/53 |
| Development_WNT signaling pathway. Part 2 | 3.30E-06 | 13/53 |
| Development_HGF-dependent inhibition of TGF-beta-induced EMT | 4.27E-06 | 12/32 |
| Development_PIP3 signaling in cardiac myocytes | 5.02E-06 | 12/47 |
| Transcription_Role of Akt in hypoxia induced HIF1 activation | 7.17E-06 | 9/27 |
| Stem cells_Inhibition of Hedgehog signaling in medulloblastoma stem cells | 7.42E-06 | 11/41 |
| Development_Regulation of CDK5 in CNS | 1.01E-05 | 9/28 |
| Development_A2A receptor signaling | 1.22E-05 | 11/43 |
| Development_Ligand-independent activation of ESR1 and ESR2 | 1.55E-05 | 11/44 |
| Development_Thyroliberin signaling | 1.73E-05 | 13/61 |
| Translation _Regulation activity of EIF4F | 1.90E-05 | 12/53 |
| Transcription_Androgen Receptor nuclear signaling | 1.96E-05 | 11/45 |
| Signal transduction_Calcium signaling | 1.96E-05 | 11/45 |
| Development_Thrombopoietin-regulated cell processes | 1.96E-05 | 11/45 |
| Development_Gastrin in cell growth and proliferation | 2.08E-05 | 13/62 |
| PGE2 pathways in cancer | 2.33E-05 | 12/54 |
| Immune response_IL-7 signaling in T lymphocytes | 2.34E-05 | 10/38 |
| Cytoskeleton remodeling_Role of PDGFs in cell migration | 2.35E-05 | 8/24 |
| Development_GDNF family signaling | 2.45E-05 | 11/46 |
| Immune response_IL-3 activation and signaling pathway | 2.54E-05 | 9/31 |
| Immune response_ETV3 affect on CSF1-promoted macrophage differentiation | 2.54E-05 | 9/31 |
| Stem cells_Embryonal epaxial myogenesis | 2.54E-05 | 9/31 |
| Immune response_IL-15 signaling | 2.98E-05 | 13/64 |
| Transcription_Receptor-mediated HIF regulation | 2.99E-05 | 10/39 |
| Development_Leptin signaling via JAK/STAT and MAPK cascades | 3.30E-05 | 8/25 |
| Apoptosis and survival_Apoptotic Activin A signaling | 3.30E-05 | 8/25 |
| Stem cells_Cooperation between Hedgehog, IGF-2 and HGF signaling pathways in medulloblastoma stem cells | 3.37E-05 | 9/32 |
| Muscle contraction_Relaxin signaling pathway | 3.76E-05 | 11/48 |
| Stem cells_Response to hypoxia in glioblastoma stem cells | 3.80E-05 | 10/40 |
| Development_WNT signaling pathway. Part 1. Degradation of beta-catenin in the absence WNT signaling | 5.42E-05 | 7/20 |
| Immune response _CCR3 signaling in eosinophils | 5.46E-05 | 14/77 |
| Apoptosis and survival_HTR1A signaling | 5.63E-05 | 11/50 |
| Development_NOTCH1-mediated pathway for NF-KB activity modulation | 5.71E-05 | 9/34 |
| Immune response_Neurotensin-induced activation of IL-8 in colonocytes | 5.98E-05 | 10/42 |
| Apoptosis and survival_BAD phosphorylation | 5.98E-05 | 10/42 |
| Stem cells_Oligodendrocyte differentiation from adult stem cells | 7.33E-05 | 9/35 |
| Immune response_IL-7 signaling in B lymphocytes | 7.42E-05 | 10/43 |
| Development_Membrane-bound ESR1: interaction with growth factors signaling | 7.42E-05 | 10/43 |
| Development_Notch Signaling Pathway | 7.42E-05 | 10/43 |
| Signal transduction_AKT signaling | 7.42E-05 | 10/43 |
| Immune response_HTR2A-induced activation of cPLA2 | 7.42E-05 | 10/43 |
| Cytoskeleton remodeling_Cytoskeleton remodeling | 1.05E-04 | 16/102 |
| Immune response_IL-6 signaling pathway | 1.08E-04 | 8/29 |
| Reproduction_GnRH signaling | 1.08E-04 | 13/72 |
| Neurophysiological process_Glutamate regulation of Dopamine D1A receptor signaling | 1.12E-04 | 10/45 |
| Immune response_Oncostatin M signaling via MAPK in human cells | 1.17E-04 | 9/37 |
| Cell adhesion_Role of tetraspanins in the integrin-mediated cell adhesion | 1.17E-04 | 9/37 |
| Development_MAG-dependent inhibition of neurite outgrowth | 1.17E-04 | 9/37 |
| Muscle contraction_Regulation of eNOS activity in endothelial cells | 1.36E-04 | 12/64 |
| Signal transduction_PTEN pathway | 1.36E-04 | 10/46 |
| Regulation of lipid metabolism_Regulation of lipid metabolism via LXR, NF-Y and SREBP | 1.46E-04 | 9/38 |
| Development_Gastrin in differentiation of the gastric mucosa | 1.46E-04 | 9/38 |
| Development_Delta- and kappa-type opioid receptors signaling via beta-arrestin | 1.49E-04 | 7/23 |
| Cytoskeleton remodeling_Regulation of actin cytoskeleton by Rho GTPases | 1.49E-04 | 7/23 |
| Development_HGF signaling pathway | 1.65E-04 | 10/47 |
| Stem cells_BMP7 in brown adipocyte differentiation | 1.81E-04 | 9/39 |
| Cell adhesion_Integrin-mediated cell adhesion and migration | 1.98E-04 | 10/48 |
| Proteolysis_Role of Parkin in the Ubiquitin-Proteasomal Pathway | 2.00E-04 | 7/24 |
| Development_GDNF signaling | 2.00E-04 | 7/24 |
| Stem cells_Pancreatic cancer stem cells in tumor metastasis | 2.23E-04 | 9/40 |
| Development_Neurotrophin family signaling | 2.23E-04 | 9/40 |
| Development_PDGF signaling via STATs and NF-kB | 2.29E-04 | 8/32 |
| Development_Role of IL-8 in angiogenesis | 2.32E-04 | 11/58 |
| Neurophysiological process_Netrin-1 in regulation of axon guidance | 2.72E-04 | 9/41 |
| Normal and pathological TGF-beta-mediated regulation of cell proliferation | 2.89E-04 | 8/33 |
| Neurophysiological process_NMDA-dependent postsynaptic long-term potentiation in CA1 hippocampal neurons | 3.24E-04 | 13/80 |
| Signal transduction_Erk Interactions: Inhibition of Erk | 3.60E-04 | 8/34 |
| G-protein signaling_RhoA regulation pathway | 3.60E-04 | 8/34 |
| Development_CNTF receptor signaling | 3.60E-04 | 8/34 |
| Chemotaxis_CXCR4 signaling pathway | 3.60E-04 | 8/34 |
| Development_ACM2 and ACM4 activation of ERK | 3.98E-04 | 9/43 |
| Signal transduction_JNK pathway | 3.98E-04 | 9/43 |
| Immune response_Oncostatin M signaling via MAPK in mouse cells | 4.45E-04 | 8/35 |
| Development_TGF-beta-dependent induction of EMT via SMADs | 4.45E-04 | 8/35 |
| Development_Growth hormone signaling via STATs and PLC/IP3 | 4.45E-04 | 8/35 |
| Stem cells_WNT and Notch signaling in early cardiac myogenesis | 4.45E-04 | 8/35 |
| Neurophysiological process_EphB receptors in dendritic spine morphogenesis and synaptogenesis | 4.45E-04 | 8/35 |
| Immune response_Signaling pathway mediated by IL-6 and IL-1 | 4.46E-04 | 7/27 |
| Stem cells_Insulin, IGF-1 and TNF-alpha in brown adipocyte differentiation | 4.64E-04 | 10/53 |
| Immune response_IL-4 signaling pathway | 4.77E-04 | 9/44 |
| Cytoskeleton remodeling_Role of Activin A in cytoskeleton remodeling | 4.90E-04 | 6/20 |
| Development_Regulation of epithelial-to-mesenchymal transition (EMT) | 5.65E-04 | 11/64 |
| Role of alpha-6/beta-4 integrins in carcinoma progression | 5.68E-04 | 9/45 |
| Development_Ligand-dependent activation of the ESR1/AP-1 pathway | 6.04E-04 | 5/14 |
| Neurophysiological process_ACM regulation of nerve impulse | 6.73E-04 | 9/46 |
| Immune response_CD137 signaling in immune cell | 7.14E-04 | 7/29 |
| Apoptosis and survival_APRIL and BAFF signaling | 8.01E-04 | 8/38 |
| Cell cycle_Regulation of G1/S transition (part 1) | 8.01E-04 | 8/38 |
| Cytoskeleton remodeling_FAK signaling | 8.46E-04 | 10/57 |
| Transcription_P53 signaling pathway | 9.61E-04 | 8/39 |
| Development_Prolactin receptor signaling | 9.74E-04 | 10/58 |
| Immune response_CCR5 signaling in macrophages and T lymphocytes | 9.74E-04 | 10/58 |
| Signal transduction_IP3 signaling | 1.09E-03 | 9/49 |
| Cytoskeleton remodeling_Fibronectin-binding integrins in cell motility | 1.10E-03 | 7/31 |
| Stem cells_Aberrant Wnt signaling in medulloblastoma stem cells | 1.11E-03 | 6/23 |
| Translation_Non-genomic (rapid) action of Androgen Receptor | 1.15E-03 | 8/40 |
| Immune response_Th1 and Th2 cell differentiation | 1.15E-03 | 8/40 |
| Cytoskeleton remodeling_Role of PKA in cytoskeleton reorganisation | 1.15E-03 | 8/40 |
| Development_GM-CSF signaling | 1.26E-03 | 9/50 |
| Development_Mu-type opioid receptor signaling via Beta-arrestin | 1.42E-03 | 6/249 |
| Transcription_Ligand-Dependent Transcription of Retinoid-Target genes | 1.62E-03 | 7/33 |
| Development_VEGF signaling and activation | 1.87E-03 | 8/43 |
| Transport_Macropinocytosis regulation by growth factors | 1.87E-03 | 10/63 |
| Development_FGFR signaling pathway | 1.93E-03 | 9/53 |
| Development_Flt3 signaling | 2.18E-03 | 8/44 |
| Development_Signaling of Beta-adrenergic receptors via Beta-arrestins | 2.21E-03 | 6/26 |
| Neurophysiological process_Dopamine D2 receptor transactivation of PDGFR in CNS | 2.21E-03 | 6/26 |
| Cell adhesion_Cadherin-mediated cell adhesion | 2.21E-03 | 6/26 |
| Immune response_IL-10 signaling pathway | 2.21E-03 | 6/26 |
| Immune response_IFN gamma signaling pathway | 2.21E-03 | 9/54 |
| Immune response_CD28 signaling | 2.21E-03 | 9/54 |
| Immune response_BCR pathway | 2.21E-03 | 9/54 |
| Development_EPO-induced Jak-STAT pathway | 2.32E-03 | 7/35 |
| Development_Angiopoietin - Tie2 signaling | 2.32E-03 | 7/35 |
| Regulation of lipid metabolism_Insulin regulation of glycogen metabolism | 2.52E-03 | 9/55 |
| Apoptosis and survival_NO synthesis and signaling | 2.52E-03 | 9/55 |
| Immune response_PGE2 signaling in immune response | 2.53E-03 | 8/45 |
| Development_EPO-induced MAPK pathway | 2.53E-03 | 8/45 |
| Neurophysiological process_GABA-A receptor life cycle | 2.71E-03 | 6/27 |
| Cell adhesion_Chemokines and adhesion | 2.73E-03 | 13/100 |
| Immune response_IL-9 signaling pathway | 2.75E-03 | 7/36 |
| wtCFTR and delta508 traffic / Clathrin coated vesicles formation (norm and CF) | 2.80E-03 | 5/19 |
| Protein folding_Membrane trafficking and signal transduction of G-alpha (i) heterotrimeric G-protein | 2.80E-03 | 5/19 |
| Development_Endothelin-1/EDNRA transactivation of EGFR | 2.92E-03 | 8/46 |
| Development_WNT5A signaling | 2.92E-03 | 8/46 |
| G-protein signaling_G-Protein alpha-12 signaling pathway | 3.24E-03 | 7/37 |
| Translation_IL-2 regulation of translation | 3.57E-03 | 5/20 |
| Immune response_Oncostatin M signaling via JAK-Stat in human cells | 3.57E-03 | 5/20 |
| Immune response_CD16 signaling in NK cells | 3.73E-03 | 10/69 |
| Stem cells_Self-renewal of adult neural stem cells | 3.85E-03 | 8/48 |
| Apoptosis and survival_nAChR in apoptosis inhibition and cell cycle progression | 3.97E-03 | 6/29 |
| Neurodisease_Parkin disorder under Parkinson disease | 3.97E-03 | 6/29 |
| Proteolysis_Putative SUMO-1 pathway | 3.97E-03 | 6/29 |
| Immune response_Delta-type opioid receptor signaling in T-cells | 3.97E-03 | 6/29 |
| Development_A3 receptor signaling | 4.39E-03 | 8/49 |
| Cytoskeleton remodeling_Integrin outside-in signaling | 4.39E-03 | 8/49 |
| Development_PACAP signaling in neural cells | 4.41E-03 | 7/39 |
| Translation _Regulation activity of EIF2 | 4.41E-03 | 7/39 |
| Transport_Clathrin-coated vesicle cycle | 4.60E-03 | 10/71 |
| Immune response_IL-4 - antiapoptotic action | 4.74E-03 | 6/30 |
| Muscle contraction_ GPCRs in the regulation of smooth muscle tone | 4.87E-03 | 11/83 |
| Immune response_Histamine signaling in dendritic cells | 4.98E-03 | 8/50 |
| Regulation of lipid metabolism_Stimulation of Arachidonic acid production by ACM receptors | 5.10E-03 | 10/72 |
| Stem cells_NOTCH1-induced self-renewal of glioblastoma stem cells | 5.10E-03 | 7/40 |
| Development_Role of Activin A in cell differentiation and proliferation | 5.10E-03 | 7/40 |
| Stem cells_FGF2-induced self-renewal of adult neural stem cells | 5.10E-03 | 7/40 |
| Reproduction_Progesterone-mediated oocyte maturation | 5.10E-03 | 7/40 |
| Transcription_PPAR Pathway | 5.17E-03 | 9/61 |
| Transcription_CREM signaling in testis | 5.55E-03 | 5/22 |
| Immune response_MIF-mediated glucocorticoid regulation | 5.55E-03 | 5/22 |
| Cytoskeleton remodeling_CDC42 in cellular processes | 5.55E-03 | 5/22 |
| Stem cells_Neovascularization of glioblastoma in response to hypoxia | 5.62E-03 | 6/31 |
| Membrane-bound ESR1: interaction with G-proteins signaling | 5.63E-03 | 8/51 |
| Some pathways of EMT in cancer cells | 5.63E-03 | 8/51 |
| Development_GH-RH signaling | 5.87E-03 | 7/41 |
| Stem cells_Regulation of lung epithelial progenitor cell differentiation | 5.87E-03 | 7/41 |
| Signal transduction_Activation of PKC via G-Protein coupled receptor | 6.35E-03 | 8/52 |
| Immune response_T cell receptor signaling pathway | 6.35E-03 | 8/52 |
| G-protein signaling_Proinsulin C-peptide signaling | 6.35E-03 | 8/52 |
| Development_EGFR signaling pathway | 6.41E-03 | 9/63 |
| Translation_Insulin regulation of translation | 6.72E-03 | 7/42 |
| Development_Growth hormone signaling via PI3K/AKT and MAPK cascades | 6.72E-03 | 7/42 |
| Development_ERK5 in cell proliferation and neuronal survival | 6.78E-03 | 5/23 |
| Proteolysis_Putative ubiquitin pathway | 6.78E-03 | 5/23 |
| Apoptosis and survival_Beta-2 adrenergic receptor anti-apoptotic action | 6.78E-03 | 5/23 |
| Immune response_IL-15 signaling via JAK-STAT cascade | 6.78E-03 | 5/23 |
| G-protein signaling_Cross-talk between Ras-family GTPases | 6.78E-03 | 5/23 |
| Apoptosis and survival_Endoplasmic reticulum stress response pathway | 7.14E-03 | 8/53 |
| Development_A1 receptor signaling | 7.14E-03 | 8/53 |
| Beta-2 adrenergic-dependent CFTR expression | 7.16E-03 | 4/15 |
| Stem cells_Aberrant Hedgehog signaling in medulloblastoma stem cells | 7.16E-03 | 4/15 |
| Regulation of lipid metabolism_Insulin regulation of fatty acid methabolism | 7.59E-03 | 11/88 |
| Development_EDG3 signaling pathway | 7.67E-03 | 7/43 |
| Apoptosis and survival_Anti-apoptotic action of Gastrin | 7.67E-03 | 7/43 |
| Development_EDG1 signaling via beta-arrestin | 7.71E-03 | 6/33 |
| Immune response_IL-22 signaling pathway | 7.71E-03 | 6/33 |
| Translation_Opioid receptors in regulation of translation | 8.19E-03 | 5/24 |
| Development_Activation of Erk by ACM1, ACM3 and ACM5 | 8.70E-03 | 7/44 |
| Cell adhesion_Alpha-4 integrins in cell migration and adhesion | 8.95E-03 | 6/34 |
| Apoptosis and survival_Role of CDK5 in neuronal death and survival | 8.95E-03 | 6/34 |
| Apoptosis and survival_Cytoplasmic/mitochondrial transport of proapoptotic proteins Bid, Bmf and Bim | 8.95E-03 | 6/34 |
| Immune response_Role of the Membrane attack complex in cell survival | 8.95E-03 | 6/34 |
| Immune response_CXCR4 signaling via second messenger | 8.95E-03 | 6/34 |
| G-protein signaling_G-Protein alpha-q signaling cascades | 8.95E-03 | 6/34 |
| Development_Role of CDK5 in neuronal development | 8.95E-03 | 6/34 |
| Immune response_IL-23 signaling pathway | 9.79E-03 | 5/25 |
| Development_Angiotensin signaling via beta-Arrestin | 9.79E-03 | 5/25 |
| Transcription_Transcription regulation of aminoacid metabolism | 9.79E-03 | 5/25 |
| Regulation of lipid metabolism_Regulation of lipid metabolism by niacin and isoprenaline | 9.84E-03 | 7/45 |
| Cell adhesion_Histamine H1 receptor signaling in the interruption of cell barrier integrity | 9.84E-03 | 7/45 |
| Muscle contraction_ACM regulation of smooth muscle contraction | 9.94E-03 | 8/56 |
| Immune response_Lipoxins and Resolvin E1 inhibitory action on neutrophil functions | 1.03E-02 | 6/35 |
| Hypoxia-induced EMT in cancer and fibrosis | 1.03E-02 | 3/9 |
| Cardiac Hypertrophy_Ca(2+)-dependent NF-AT signaling in Cardiac Hypertrophy | 1.10E-02 | 8/57 |
| Development_G-Proteins mediated regulation MARK-ERK signaling | 1.11E-02 | 7/46 |
| Development_TGF-beta-dependent induction of EMT via RhoA, PI3K and ILK. | 1.11E-02 | 7/46 |
| Immune response_MIF - the neuroendocrine-macrophage connector | 1.11E-02 | 6/46 |
| G-protein signaling_Rap2A regulation pathway | 1.15E-02 | 4/17 |
| Immune response_Regulation of T cell function by CTLA-4 | 1.18E-02 | 6/36 |
| G-protein signaling_RAC1 in cellular process | 1.18E-02 | 6/36 |
| Regulation of CFTR activity (norm and CF) | 1.22E-02 | 8/58 |
| Regulation of lipid metabolism_Insulin signaling:generic cascades | 1.24E-02 | 6/47 |
| Neurophysiological process_Circadian rhythm | 1.24E-02 | 6/47 |
| Development_Hedgehog and PTH signaling pathways in bone and cartilage development | 1.35E-02 | 6/37 |
| Development_Beta-adrenergic receptors transactivation of EGFR | 1.35E-02 | 6/37 |
| G-protein signaling_G-Protein alpha-i signaling cascades | 1.36E-02 | 5/27 |
| Apoptosis and survival_Anti-apoptotic TNFs/NF-kB/IAP pathway | 1.36E-02 | 5/27 |
| Possible pathway of TGF-beta 1-dependent inhibition of CFTR expression | 1.36E-02 | 5/27 |
| Blood coagulation_GPCRs in platelet aggregation | 1.38E-02 | 9/71 |
| Immune response_Histamine H1 receptor signaling in immune response | 1.39E-02 | 7/48 |
| Regulation of metabolism_Triiodothyronine and Thyroxine signaling | 1.39E-02 | 7/48 |
| Immune response_Oncostatin M signaling via JAK-Stat in mouse cells | 1.41E-02 | 4/18 |
| Development_Mu-type opioid receptor signaling | 1.53E-02 | 6/38 |
| Development_Mu-type opioid receptor regulation of proliferation | 1.59E-02 | 5/28 |
| Regulation of lipid metabolism_Regulation of fatty acid synthase activity in hepatocytes | 1.71E-02 | 4/19 |
| Development_EDNRB signaling | 1.72E-02 | 7/50 |
| Development_A2B receptor: action via G-protein alpha s | 1.72E-02 | 7/50 |
| Development_ERBB-family signaling | 1.73E-02 | 6/39 |
| Cell adhesion_PLAU signaling | 1.73E-02 | 6/39 |
| Cell cycle_Role of SCF complex in cell cycle regulation | 1.83E-02 | 5/29 |
| Mucin expression in CF via TLRs, EGFR signaling pathways | 1.90E-02 | 7/51 |
| Chemotaxis_Inhibitory action of lipoxins on IL-8- and Leukotriene B4-induced neutrophil migration | 1.90E-02 | 7/51 |
| Chemotaxis_Leukocyte chemotaxis | 1.93E-02 | 9/75 |
| Inhibitory action of Lipoxins and Resolvin E1 on neutrophil functions | 1.95E-02 | 6/40 |
| Development_FGF2-dependent induction of EMT | 2.05E-02 | 4/20 |
| Cell adhesion_ECM remodeling | 2.10E-02 | 7/52 |
| Stem cells_mGluR3 signaling in glioblastoma stem cells | 2.10E-02 | 7/52 |
| Development_Beta-adrenergic receptors signaling via cAMP | 2.10E-02 | 7/52 |
| Neurophysiological process_Mu-type opioid receptor-mediated analgesia | 2.11E-02 | 5/30 |
| Immune response_CD40 signaling | 2.13E-02 | 8/64 |
| Apoptosis and survival_Anti-apoptotic TNFs/NF-kB/Bcl-2 pathway | 2.18E-02 | 6/41 |
| Development_VEGF-family signaling | 2.18E-02 | 6/41 |
| Apoptosis and survival_Lymphotoxin-beta receptor signaling | 2.18E-02 | 6/41 |
| Development_Endothelin-1/EDNRA signaling | 2.31E-02 | 7/53 |
| Stem cells_Dopamine-induced transactivation of EGFR in SVZ neural stem cells | 2.40E-02 | 5/31 |
| Neurophysiological process_HTR1A receptor signaling in neuronal cells | 2.43E-02 | 6/42 |
| Immune response_PIP3 signaling in B lymphocytes | 2.43E-02 | 6/42 |
| Development_Angiotensin signaling via PYK2 | 2.70E-02 | 6/43 |
| Neurophysiological process_Melatonin signaling | 2.70E-02 | 6/43 |
| Development_Transcription regulation of granulocyte development | 2.72E-02 | 5/32 |
| Development_Angiotensin signaling via STATs | 2.72E-02 | 5/32 |
| Immune response_Fc epsilon RI pathway | 2.78E-02 | 7/55 |
| Cell cycle_Sister chromatid cohesion | 2.85E-02 | 4/22 |
| Development_Thrombopoetin signaling via JAK-STAT pathway | 2.85E-02 | 4/22 |
| Immune response_IL-5 signalling | 2.99E-02 | 6/44 |
| Development_Angiotensin activation of ERK | 3.07E-02 | 3/33 |
| Cytoskeleton remodeling_Thyroliberin in cytoskeleton remodeling | 3.07E-02 | 3/33 |
| Cell cycle_ESR1 regulation of G1/S transition | 3.07E-02 | 3/33 |
| Immune response_Inhibitory action of Lipoxins on pro-inflammatory TNF-alpha signaling | 3.30E-02 | 6/45 |
| Immune response_Fc gamma R-mediated phagocytosis in macrophages | 3.30E-02 | 6/45 |
| Inhibitory action of Lipoxins on neutrophil migration | 3.30E-02 | J7/57 |
| Inhibitory action of Lipoxin A4 on PDGF, EGF and LTD4 signaling | 3.45E-02 | 5/34 |
| Mucin expression in CF via IL-6, IL-17 signaling pathways | 3.45E-02 | 5/34 |
| Immune response_NF-AT signaling and leukocyte interactions | 3.63E-02 | 6/46 |
| CFTR folding and maturation (norm and CF) | 3.67E-02 | 3/14 |
| Development_Dopamine D2 receptor transactivation of EGFR | 3.80E-02 | 4/24 |
| Immune response_IL-27 signaling pathway | 3.80E-02 | 4/24 |
| Cell adhesion_Plasmin signaling | 3.85E-02 | 5/35 |
| G-protein signaling_EDG5 signaling | 3.85E-02 | 5/35 |
| Development_Lipoxin inhibitory action on PDGF, EGF and LTD4 signaling | 3.85E-02 | 5/35 |
| Transcription_Role of VDR in regulation of genes involved in osteoporosis | 3.89E-02 | 4/59 |
| Development_Beta-adrenergic receptors regulation of ERK | 3.97E-02 | 4/47 |
| Development_TGF-beta-dependent induction of EMT via MAPK | 3.97E-02 | 4/47 |
| Development_PDGF signaling via MAPK cascades | 3.97E-02 | 4/47 |
| Development_Activation of ERK by Kappa-type opioid receptor | 4.28E-02 | 5/36 |
| Cell adhesion_Tight junctions | 4.28E-02 | 5/36 |
| Transport_RAB5A regulation pathway | 4.33E-02 | 4/25 |
| Cytokine production by Th17 cells in CF (Mouse model) | 4.73E-02 | 6/49 |
| Immune response_Bacterial infections in normal airways | 4.73E-02 | 6/49 |
| Stem cells_Beta adrenergic receptors in brown adipocyte differentiation | 4.74E-02 | 3/37 |
| Cytoskeleton remodeling_ACM3 and ACM4 in keratinocyte migration | 4.74E-02 | 3/37 |
| G-protein signaling_H-RAS regulation pathway | 4.74E-02 | 3/37 |
| Development_Alpha-2 adrenergic receptor activation of ERK | 4.89E-02 | 7/62 |
| G-protein signaling_Ras family GTPases in kinase cascades (scheme) | 4.91E-02 | 4/26 |
| Development_Cross-talk between VEGF and Angiopoietin 1 signaling pathways | 4.91E-02 | 4/26 |
| Development_EDG5 and EDG3 in cell proliferation and differentiation | 4.91E-02 | 4/26 |
