# Supplementary material for: Involvement of Histone Acetylation of Sox17 and Foxa2 Promoters during Mouse Definitive Endoderm Differentiation Revealed by MicroRNA Profiling
Source: PLoS One. 2011 Nov 23;6(11):e27965. doi: 10.1371/journal.pone.0027965 (PMC3223193; doi:10.1371/journal.pone.0027965)
Supplement: Table S6 — Enriched pathways of synergistically regulated miRNAs at DE maturation stages. GeneGo pathway analysis of putative targeted genes was performed on the synergistically regulated miRNAs at the DE maturation stage. 252 pathways were significantly enriched. (DOC) [file pone.0027965.s014.doc]

Table S6.enriched pathways of synergistically regulated miRNAs at maturation stages.

| Name | pValue | Network objects |
| --- | --- | --- |
| Transcription_Sin3 and NuRD in transcription regulation | 6.79E-11 | 16/38 |
| Development_Thrombopoietin-regulated cell processes | 1.25E-08 | 15/45 |
| Cell cycle_ESR1 regulation of G1/S transition | 1.22E-07 | 12/33 |
| Stem cells_Self-renewal of adult neural stem cells | 2.56E-07 | 14/48 |
| Transcription_Ligand-dependent activation of the ESR1/SP pathway | 3.76E-07 | 11/30 |
| Cardiac Hypertrophy_NF-AT signaling in Cardiac Hypertrophy | 4.70E-07 | 16/65 |
| Development_PDGF signaling via STATs and NF-kB | 8.00E-07 | 11/32 |
| Signal transduction_Activin A signaling regulation | 1.14E-06 | 11/33 |
| Development_Role of HDAC and calcium/calmodulin-dependent kinase (CaMK) in control of skeletal myogenesis | 1.25E-06 | 14/54 |
| Development_HGF signaling pathway | 1.35E-06 | 13/47 |
| Stem cells_Dopamine-induced expression of CNTF in adult neurogenesis | 1.51E-06 | 9/22 |
| G-protein signaling_RhoA regulation pathway | 1.60E-06 | 11/34 |
| Stem cells_WNT and Notch signaling in early cardiac myogenesis | 2.21E-06 | 11/35 |
| Immune response_Function of MEF2 in T lymphocytes | 2.91E-06 | 13/50 |
| Development_Membrane-bound ESR1: interaction with growth factors signaling | 3.12E-06 | 12/43 |
| Development_Notch Signaling Pathway | 3.12E-06 | 12/43 |
| Development_IGF-1 receptor signaling | 3.70E-06 | 13/51 |
| Transcription_CREB pathway | 4.07E-06 | 12/44 |
| Immune response_ETV3 affect on CSF1-promoted macrophage differentiation | 4.96E-06 | 10/31 |
| Signal transduction_Calcium signaling | 5.27E-06 | 12/45 |
| Development_PIP3 signaling in cardiac myocytes | 8.62E-06 | 12/47 |
| Muscle contraction_Regulation of eNOS activity in endothelial cells | 1.09E-05 | 14/64 |
| Stem cells_Regulation of lung epithelial progenitor cell differentiation | 1.23E-05 | 11/41 |
| Immune response_IL-7 signaling in B lymphocytes | 2.01E-05 | 11/43 |
| Immune response_IL-6 signaling pathway | 2.12E-05 | 9/29 |
| Stem cells_Notch signaling in medulloblastoma stem cells | 2.12E-05 | 9/29 |
| Cell adhesion_ECM remodeling | 2.62E-05 | 12/52 |
| Neurophysiological process_Receptor-mediated axon growth repulsion | 3.20E-05 | 11/45 |
| Development_FGFR signaling pathway | 3.22E-05 | 12/53 |
| Cytoskeleton remodeling_Role of PDGFs in cell migration | 3.44E-05 | 8/24 |
| Cell cycle_Regulation of G1/S transition (part 1) | 3.68E-05 | 10/38 |
| Immune response_IFN gamma signaling pathway | 3.93E-05 | 12/54 |
| Signal transduction_PTEN pathway | 3.99E-05 | 11/46 |
| Transcription_Receptor-mediated HIF regulation | 4.70E-05 | 10/39 |
| Development_PDGF signaling via MAPK cascades | 4.95E-05 | 11/47 |
| Development_HGF-dependent inhibition of TGF-beta-induced EMT | 5.10E-05 | 9/32 |
| Development_EGFR signaling via small GTPases | 5.10E-05 | 9/32 |
| Cytoskeleton remodeling_Cytoskeleton remodeling | 5.65E-05 | 17/102 |
| Stem cells_NOTCH1-induced self-renewal of glioblastoma stem cells | 5.95E-05 | 10/40 |
| Stem cells_FGF2-induced self-renewal of adult neural stem cells | 5.95E-05 | 10/40 |
| Regulation of metabolism_Triiodothyronine and Thyroxine signaling | 6.09E-05 | 11/48 |
| Cell adhesion_Role of CDK5 in cell adhesion | 6.14E-05 | 5/9 |
| Cell cycle_Regulation of G1/S transition (part 2) | 6.61E-05 | 8/26 |
| Normal and pathological TGF-beta-mediated regulation of cell proliferation | 6.66E-05 | 11/33 |
| Immune response_IL-2 activation and signaling pathway | 7.46E-05 | 11/49 |
| Signal transduction_IP3 signaling | 7.46E-05 | 11/49 |
| Signal transduction_PKA signaling | 1.10E-04 | 11/51 |
| Development_TGF-beta-dependent induction of EMT via SMADs | 1.10E-04 | 9/35 |
| Development_Growth hormone signaling via STATs and PLC/IP3 | 1.10E-04 | 9/35 |
| Immune response_Gastrin in inflammatory response | 1.17E-04 | 13/69 |
| Transcription_PPAR Pathway | 1.38E-04 | 12/61 |
| Development_Flt3 signaling | 1.42E-04 | 10/44 |
| Cytoskeleton remodeling_TGF, WNT and cytoskeletal remodeling | 1.67E-04 | 17/111 |
| Transcription_Androgen Receptor nuclear signaling | 1.73E-04 | 10/45 |
| Cell adhesion_Ephrins signaling | 1.73E-04 | 10/45 |
| Development_EPO-induced MAPK pathway | 1.73E-04 | 10/45 |
| Immune response_Oncostatin M signaling via MAPK in human cells | 1.75E-04 | 9/37 |
| G-protein signaling_H-RAS regulation pathway | 1.75E-04 | 9/37 |
| Reproduction_GnRH signaling | 1.83E-04 | 13/72 |
| PGE2 pathways in cancer | 1.89E-04 | 11/54 |
| Development_Endothelin-1/EDNRA transactivation of EGFR | 2.10E-04 | 10/46 |
| Signal transduction_cAMP signaling | 2.18E-04 | 9/38 |
| Regulation of lipid metabolism_Regulation of lipid metabolism via LXR, NF-Y and SREBP | 2.18E-04 | 9/38 |
| Development_Regulation of epithelial-to-mesenchymal transition (EMT) | 2.23E-04 | 12/64 |
| Development_TGF-beta-dependent induction of EMT via MAPK | 2.54E-04 | 10/47 |
| Translation _Regulation activity of EIF2 | 2.70E-04 | 9/39 |
| Development_Role of Activin A in cell differentiation and proliferation | 3.31E-04 | 9/40 |
| G-protein signaling_K-RAS regulation pathway | 3.67E-04 | 7/25 |
| Transcription_Ligand-Dependent Transcription of Retinoid-Target genes | 4.12E-04 | 8/33 |
| Development_TGF-beta receptor signaling | 4.31E-04 | 10/50 |
| Development_GM-CSF signaling | 4.31E-04 | 10/50 |
| Neurophysiological process_Dopamine D2 receptor transactivation of PDGFR in CNS | 4.78E-04 | 7/26 |
| Cell adhesion_Cadherin-mediated cell adhesion | 4.78E-04 | 7/26 |
| Development_Alpha-1 adrenergic receptors signaling via cAMP | 4.78E-04 | 6/19 |
| Regulation of lipid metabolism_Regulation of fatty acid synthase activity in hepatocytes | 4.78E-04 | 6/19 |
| Translation_Insulin regulation of translation | 4.87E-04 | 9/42 |
| Membrane-bound ESR1: interaction with G-proteins signaling | 5.09E-04 | 10/51 |
| Some pathways of EMT in cancer cells | 5.09E-04 | 10/51 |
| Development_CNTF receptor signaling | 5.13E-04 | 8/34 |
| Chemotaxis_CXCR4 signaling pathway | 5.13E-04 | 8/34 |
| Development_NOTCH1-mediated pathway for NF-KB activity modulation | 5.13E-04 | 8/34 |
| Development_Thyroliberin signaling | 5.75E-04 | 11/61 |
| Development_ACM2 and ACM4 activation of ERK | 5.85E-04 | 9/43 |
| Signal transduction_AKT signaling | 5.85E-04 | 9/43 |
| Immune response_Oncostatin M signaling via MAPK in mouse cells | 6.32E-04 | 8/35 |
| Neurophysiological process_EphB receptors in dendritic spine morphogenesis and synaptogenesis | 6.32E-04 | 8/35 |
| Cytoskeleton remodeling_Role of Activin A in cytoskeleton remodeling | 6.50E-04 | 6/20 |
| Immune response_Oncostatin M signaling via JAK-Stat in human cells | 6.50E-04 | 6/20 |
| Development_Gastrin in cell growth and proliferation | 6.64E-04 | 11/62 |
| Development_Ligand-independent activation of ESR1 and ESR2 | 6.99E-04 | 9/44 |
| Translation _Regulation activity of EIF4F | 7.02E-04 | 10/53 |
| Stem cells_Insulin, IGF-1 and TNF-alpha in brown adipocyte differentiation | 7.02E-04 | 10/53 |
| Development_EGFR signaling pathway | 7.64E-04 | 11/63 |
| Transport_Macropinocytosis regulation by growth factors | 7.64E-04 | 11/63 |
| Immune response_BCR pathway | 8.18E-04 | 10/54 |
| Cell cycle_Cell cycle (generic schema) | 8.67E-04 | 6/21 |
| Immune response_Delta-type opioid receptor signaling in T-cells | 9.76E-04 | 7/29 |
| Hypoxia-induced EMT in cancer and fibrosis | 1.05E-03 | 4/9 |
| Immune response_IL-7 signaling in T lymphocytes | 1.13E-03 | 8/38 |
| Development_Gastrin in differentiation of the gastric mucosa | 1.13E-03 | 8/38 |
| Transcription_Role of heterochromatin protein 1 (HP1) family in transcriptional silencing | 1.14E-03 | 6/22 |
| Cytoskeleton remodeling_FAK signaling | 1.27E-03 | 10/57 |
| Cell adhesion_Integrin-mediated cell adhesion and migration | 1.35E-03 | 9/48 |
| Immune response_CCR5 signaling in macrophages and T lymphocytes | 1.45E-03 | 10/58 |
| Stem cells_Dopamine-induced transactivation of EGFR in SVZ neural stem cells | 1.49E-03 | 7/31 |
| Immune response_IL-3 activation and signaling pathway | 1.49E-03 | 7/31 |
| Cytoskeleton remodeling_Reverse signaling by ephrin B | 1.49E-03 | 7/31 |
| Development_Transactivation of PDGFR in non-neuronal cells by Dopamine D2 receptor | 1.49E-03 | 7/31 |
| Cell adhesion_Chemokines and adhesion | 1.52E-03 | 14/100 |
| G-protein signaling_RhoB regulation pathway | 1.53E-03 | 5/16 |
| Stem cells_Response to hypoxia in glioblastoma stem cells | 1.61E-03 | 8/40 |
| Stem cells_Pancreatic cancer stem cells in tumor metastasis | 1.61E-03 | 8/40 |
| Development_Neurotrophin family signaling | 1.61E-03 | 8/40 |
| Apoptosis and survival_HTR1A signaling | 1.82E-03 | 9/50 |
| Development_Dopamine D2 receptor transactivation of EGFR | 1.86E-03 | 6/24 |
| Transport_Clathrin-coated vesicle cycle | 2.11E-03 | 11/71 |
| G-protein signaling_N-RAS regulation pathway | 2.19E-03 | 7/33 |
| Development_Leptin signaling via JAK/STAT and MAPK cascades | 2.33E-03 | 6/25 |
| Stem cells_mGluR3 signaling in glioblastoma stem cells | 2.42E-03 | 9/52 |
| ENaC regulation in airways (normal and CF) | 2.42E-03 | 9/52 |
| Development_Angiotensin signaling via PYK2 | 2.61E-03 | 8/43 |
| Signal transduction_JNK pathway | 2.61E-03 | 8/43 |
| Development_A2A receptor signaling | 2.61E-03 | 8/43 |
| Immune response_Oncostatin M signaling via JAK-Stat in mouse cells | 2.72E-03 | 5/18 |
| Transport_RAN regulation pathway | 2.72E-03 | 5/18 |
| Immune response_IL-10 signaling pathway | 2.88E-03 | 6/26 |
| Development_Activation of Erk by ACM1, ACM3 and ACM5 | 3.03E-03 | 8/44 |
| Immune response_IL-5 signalling | 3.03E-03 | 8/44 |
| Immune response_IL-15 signaling | 3.11E-03 | 10/64 |
| Development_EPO-induced Jak-STAT pathway | 3.13E-03 | 7/35 |
| Cell adhesion_Histamine H1 receptor signaling in the interruption of cell barrier integrity | 3.51E-03 | 8/45 |
| Blood coagulation_GPVI-dependent platelet activation | 3.59E-03 | 9/55 |
| Regulation of lipid metabolism_Insulin regulation of glycogen metabolism | 3.59E-03 | 9/55 |
| G-protein signaling_RAC1 in cellular process | 3.70E-03 | 7/36 |
| Development_G-Proteins mediated regulation MARK-ERK signaling | 4.04E-03 | 8/46 |
| Development_GDNF family signaling | 4.04E-03 | 8/46 |
| Development_TGF-beta-dependent induction of EMT via RhoA, PI3K and ILK. | 4.04E-03 | 8/46 |
| Development_Mu-type opioid receptor regulation of proliferation | 4.28E-03 | 6/28 |
| Development_Delta-type opioid receptor signaling via G-protein alpha-14 | 4.28E-03 | 6/28 |
| Stem cells_Beta adrenergic receptors in brown adipocyte differentiation | 4.34E-03 | 7/37 |
| Development_Hedgehog and PTH signaling pathways in bone and cartilage development | 4.34E-03 | 7/37 |
| Translation_IL-2 regulation of translation | 4.48E-03 | 5/20 |
| Regulation of lipid metabolism_Insulin signaling:generic cascades | 4.63E-03 | 8/47 |
| Cell cycle_Role of SCF complex in cell cycle regulation | 5.15E-03 | 6/29 |
| Development_SSTR1 in regulation of cell proliferation and migration | 5.15E-03 | 6/29 |
| Development_Role of IL-8 in angiogenesis | 5.17E-03 | 9/58 |
| Development_Prolactin receptor signaling | 5.17E-03 | 9/58 |
| Neurophysiological process_NMDA-dependent postsynaptic long-term potentiation in CA1 hippocampal neurons | 5.44E-03 | 11/80 |
| Development_ERBB-family signaling | 5.87E-03 | 7/39 |
| Stem cells_BMP7 in brown adipocyte differentiation | 5.87E-03 | 7/39 |
| Regulation of lipid metabolism_RXR-dependent regulation of lipid metabolism via PPAR, RAR and VDR | 6.13E-03 | 6/30 |
| Immune response_IL-4 - antiapoptotic action | 6.13E-03 | 6/30 |
| Development_Ligand-dependent activation of the ESR1/AP-1 pathway | 6.61E-03 | 4/14 |
| Cell cycle_Nucleocytoplasmic transport of CDK/Cyclins | 6.61E-03 | 4/14 |
| Translation_Non-genomic (rapid) action of Androgen Receptor | 6.78E-03 | 7/40 |
| Development_EDNRB signaling | 6.81E-03 | 8/50 |
| Development_Thrombopoetin signaling via JAK-STAT pathway | 6.92E-03 | 5/22 |
| Stem cells_Embryonal epaxial myogenesis | 7.24E-03 | 6/31 |
| Development_ERK5 in cell proliferation and neuronal survival | 8.44E-03 | 5/23 |
| Development_Delta- and kappa-type opioid receptors signaling via beta-arrestin | 8.44E-03 | 5/23 |
| Development_EGFR signaling via PIP3 | 8.44E-03 | 5/23 |
| Immune response_IL-15 signaling via JAK-STAT cascade | 8.44E-03 | 5/23 |
| G-protein signaling_Cross-talk between Ras-family GTPases | 8.44E-03 | 5/23 |
| Development_Transcription regulation of granulocyte development | 8.49E-03 | 6/32 |
| Beta-2 adrenergic-dependent CFTR expression | 8.61E-03 | 4/15 |
| Signal transduction_Activation of PKC via G-Protein coupled receptor | 8.65E-03 | 8/52 |
| Development_Beta-adrenergic receptors signaling via cAMP | 8.65E-03 | 8/52 |
| Development_A1 receptor signaling | 9.69E-03 | 8/53 |
| Stem cells_Differentiation of white adipocytes | 9.69E-03 | 8/53 |
| Development_Endothelin-1/EDNRA signaling | 9.69E-03 | 8/53 |
| Development_WNT signaling pathway. Part 2 | 9.69E-03 | 8/53 |
| Development_Angiotensin activation of ERK | 9.89E-03 | 6/33 |
| Development_EDG3 signaling pathway | 1.01E-02 | 6/43 |
| Development_VEGF signaling and activation | 1.01E-02 | 6/43 |
| Apoptosis and survival_Anti-apoptotic action of Gastrin | 1.01E-02 | 6/43 |
| Immune response_HTR2A-induced activation of cPLA2 | 1.01E-02 | 6/43 |
| Cell adhesion_Endothelial cell contacts by non-junctional mechanisms | 1.02E-02 | 5/24 |
| Immune response_CD28 signaling | 1.08E-02 | 8/54 |
| Apoptosis and survival_Role of CDK5 in neuronal death and survival | 1.15E-02 | 6/34 |
| Immune response_IL-4 signaling pathway | 1.15E-02 | 7/44 |
| Immune response _CCR3 signaling in eosinophils | 1.17E-02 | 10/77 |
| Immune response_Fc epsilon RI pathway | 1.21E-02 | 8/55 |
| Apoptosis and survival_Apoptotic Activin A signaling | 1.21E-02 | 5/25 |
| Regulation of lipid metabolism_Regulation of lipid metabolism by niacin and isoprenaline | 1.29E-02 | 7/45 |
| Role of alpha-6/beta-4 integrins in carcinoma progression | 1.29E-02 | 7/45 |
| Apoptosis and survival_Anti-apoptotic action of membrane-bound ESR1 | 1.32E-02 | 6/35 |
| Stem cells_Astrocyte differentiation from adult stem cells | 1.32E-02 | 6/35 |
| G-protein signaling_Ras family GTPases in kinase cascades (scheme) | 1.43E-02 | 5/26 |
| Apoptosis and survival_NGF signaling pathway | 1.43E-02 | 5/26 |
| Development_Signaling of Beta-adrenergic receptors via Beta-arrestins | 1.43E-02 | 5/26 |
| Cardiac Hypertrophy_Ca(2+)-dependent NF-AT signaling in Cardiac Hypertrophy | 1.48E-02 | 8/57 |
| Immune response_IL-9 signaling pathway | 1.51E-02 | 6/36 |
| Immune response_Regulation of T cell function by CTLA-4 | 1.51E-02 | 6/36 |
| G-protein signaling_Regulation of RAC1 activity | 1.51E-02 | 6/36 |
| Development_Beta-adrenergic receptors regulation of ERK | 1.63E-02 | 7/47 |
| Regulation of CFTR activity (norm and CF) | 1.64E-02 | 8/58 |
| G-protein signaling_G-Protein alpha-i signaling cascades | 1.68E-02 | 5/27 |
| Neurophysiological process_GABA-A receptor life cycle | 1.68E-02 | 5/27 |
| Development_Beta-adrenergic receptors transactivation of EGFR | 1.72E-02 | 6/37 |
| Stem cells_Early embryonal hypaxial myogenesis | 1.72E-02 | 6/37 |
| Immune response _Immunological synapse formation | 1.80E-02 | 8/59 |
| Muscle contraction_ GPCRs in the regulation of smooth muscle tone | 1.91E-02 | 10/83 |
| Development_Regulation of CDK5 in CNS | 1.95E-02 | 5/28 |
| Development_A3 receptor signaling | 2.01E-02 | 7/49 |
| Cytoskeleton remodeling_Integrin outside-in signaling | 2.01E-02 | 7/49 |
| Development_TGF-beta-induction of EMT via ROS | 2.04E-02 | 4/19 |
| wtCFTR and delta508 traffic / Clathrin coated vesicles formation (norm and CF) | 2.04E-02 | 4/19 |
| Development_NOTCH-induced EMT | 2.04E-02 | 4/19 |
| Development_PACAP signaling in neural cells | 2.19E-02 | 6/39 |
| Cell adhesion_PLAU signaling | 2.19E-02 | 6/39 |
| Apoptosis and survival_nAChR in apoptosis inhibition and cell cycle progression | 2.25E-02 | 5/29 |
| G-protein signaling_Rap1A regulation pathway | 2.45E-02 | 6/40 |
| Reproduction_Progesterone-mediated oocyte maturation | 2.45E-02 | 6/40 |
| DNA damage_Brca1 as a transcription regulator | 2.58E-02 | 5/30 |
| Development_Slit-Robo signaling | 2.58E-02 | 5/30 |
| Cytoskeleton remodeling_RalA regulation pathway | 2.58E-02 | 5/30 |
| Blood coagulation_GPIb-IX-V-dependent platelet activation | 2.61E-02 | 9/75 |
| G-protein signaling_Proinsulin C-peptide signaling | 2.71E-02 | 7/52 |
| Neurophysiological process_Netrin-1 in regulation of axon guidance | 2.74E-02 | 6/41 |
| Transport_Macropinocytosis | 2.76E-02 | 3/12 |
| Regulation of lipid metabolism_Insulin regulation of fatty acid methabolism | 2.76E-02 | 10/88 |
| Stem cells_Neovascularization of glioblastoma in response to hypoxia | 2.94E-02 | 5/31 |
| Translation_Translation regulation by Alpha-1 adrenergic receptors | 2.98E-02 | 7/53 |
| Immune response_Neurotensin-induced activation of IL-8 in colonocytes | 3.05E-02 | 6/42 |
| Apoptosis and survival_BAD phosphorylation | 3.05E-02 | 6/42 |
| Development_Growth hormone signaling via PI3K/AKT and MAPK cascades | 3.05E-02 | 6/42 |
| Stem cells_Cooperation between Hedgehog, IGF-2 and HGF signaling pathways in medulloblastoma stem cells | 3.32E-02 | 5/32 |
| Autophagy_Autophagy | 3.32E-02 | 5/32 |
| Development_Angiotensin signaling via STATs | 3.32E-02 | 5/32 |
| Cytoskeleton remodeling_CDC42 in cellular processes | 3.37E-02 | 4/22 |
| Cytoskeleton remodeling_RalB regulation pathway | 3.43E-02 | 3/13 |
| Immune response_IL-22 signaling pathway | 3.74E-02 | 5/33 |
| Cell adhesion_Integrin inside-out signaling | 3.88E-02 | 7/56 |
| Immune response_TREM1 signaling pathway | 3.88E-02 | 7/56 |
| Stem cells_Aberrant Wnt signaling in medulloblastoma stem cells | 3.90E-02 | 4/23 |
| Neurophysiological process_Glutamate regulation of Dopamine D1A receptor signaling | 4.11E-02 | 6/45 |
| Immune response_Inhibitory action of Lipoxins on pro-inflammatory TNF-alpha signaling | 4.11E-02 | 6/45 |
| Immune response_Fc gamma R-mediated phagocytosis in macrophages | 4.11E-02 | 6/45 |
| Development_Activation of ERK by Alpha-1 adrenergic receptors | 4.11E-02 | 6/45 |
| Immune response_CD16 signaling in NK cells | 4.16E-02 | 8/69 |
| G-protein signaling_G-Protein beta/gamma signaling cascades | 4.19E-02 | 5/34 |
| Apoptosis and survival_Cytoplasmic/mitochondrial transport of proapoptotic proteins Bid, Bmf and Bim | 4.19E-02 | 5/34 |
| Immune response_Role of the Membrane attack complex in cell survival | 4.19E-02 | 5/34 |
| G-protein signaling_G-Protein alpha-q signaling cascades | 4.19E-02 | 5/34 |
| Development_Role of CDK5 in neuronal development | 4.19E-02 | 5/34 |
| Proteolysis_Role of Parkin in the Ubiquitin-Proteasomal Pathway | 4.48E-02 | 4/24 |
| Development_GDNF signaling | 4.48E-02 | 4/24 |
| Immune response_MIF-JAB1 signaling | 4.48E-02 | 4/24 |
| Immune response_MIF - the neuroendocrine-macrophage connector | 4.51E-02 | 6/46 |
| Development_WNT5A signaling | 4.51E-02 | 6/46 |
| Development_FGF-family signaling | 4.66E-02 | 5/35 |
| Blood coagulation_GPCRs in platelet aggregation | 4.80E-02 | 8/71 |
